# Neutrophil-specific targeting of STAT3 impairs tumor progression via the expansion of cytotoxic CD8^+^ T cells

**DOI:** 10.1038/s41392-025-02363-z

**Published:** 2025-08-30

**Authors:** Irem Ozel, Guanyu Sha, Agnieszka Będzińska, Ekaterina Pylaeva, Yuliia Naumova, Ilona Thiel, Joanna Antczak, Anthony Squire, Matthias Gunzer, Gennadiy Zelinskyy, Cornelius Kürten, Stephan Lang, Carlos Silvestre-Roig, Marcin Kortylewski, Zvi Granot, Jadwiga Jablonska

**Affiliations:** 1 Department of Otorhinolaryngology, Head and Neck Surgery, University Hospital Essen, Essen, University of Duisburg-Essen, Essen, 45147 Germany; 2 Center for Translational Research and Molecular Biology of Cancer, Maria Skłodowska-Curie National Research Institute of Oncology, Gliwice, 44101 Poland; 3 Institute for Experimental Immunology and Imaging, University Hospital Essen, University of Duisburg-Essen, Essen, 45141 Germany; 4 Institute for Virology, University Hospital Essen, University of Duisburg-Essen, Essen, 45147 Germany; 5 German Cancer Consortium (DKTK) partner site Düsseldorf/Essen, Essen, 45147 Germany; 6 Institute of Experimental Pathology (ExPat), Center for Molecular Biology of Inflammation (ZMBE), University of Münster, Münster, 48149 Germany; 7 Department of Immuno-Oncology, Beckman Research Institute at City of Hope Comprehensive Cancer Center, Duarte, CA 91010 USA; 8 Center for Gene Therapy, Beckman Research Institute at City of Hope Comprehensive Cancer Center, Duarte, CA 91010 USA; 9 Department of Developmental Biology and Cancer Research, Institute for Medical Research Israel Canada, Faculty of Medicine, Hebrew University, Jerusalem, 91120 Israel

**Keywords:** Cancer therapy, Tumour immunology, Immunotherapy

## Abstract

Neutrophils have emerged as key players in tumor progression and are often associated with poor prognosis. Despite ongoing efforts to target neutrophil functions in cancer, therapeutic success has been limited. In this study, we addressed the possibility of blocking STAT3 signaling in neutrophils as a targeted therapeutic intervention in cancer. Conditional deletion of *Stat3* in a neutrophil-specific manner (*Ly6G*^*cre*^*Stat3*^*fl/fl*^ mice) significantly impaired tumor growth and metastasis in mice. Neutrophils isolated from these mice exhibited a strong antitumoral phenotype, with increased MHCII, CD80/86 and ICAM-1 expression. Immune profiling of tumors and tumor-draining lymph nodes of these mice revealed significant enrichment of CD8^+^ T cells (granzymeB^hi^, perforin^hi^ and IFN-γ^hi^) with strong cytotoxic activity. To further translate these findings to human settings, we blocked STAT3 signaling in cancer patient neutrophils via the small molecule inhibitor LLL12 and assessed its effects on patient-derived tumor explants. In agreement with the in vivo mouse data, we observed the expansion and activation of cytotoxic CD8^+^ T cells in such explants. To test the therapeutic applicability of STAT3 targeting, we utilized myeloid cell-selective STAT3 antisense oligonucleotide (CpG-STAT3ASO) to target neutrophils in vivo in tumor-bearing mice. Consistent with previous results, neutrophil-specific STAT3 knockdown impaired tumor growth and enhanced cytotoxic T cell activity in the tumors and tumor-draining lymph nodes of treated mice. These findings highlight STAT3 signaling as a deleterious pathway supporting the protumoral activity of neutrophils and suggest that neutrophil-targeted STAT3 inhibition is a promising opportunity for cancer immunotherapy, providing novel insights into targeted therapeutic avenues.

## Introduction

Harnessing the innate immune system, particularly the functional plasticity of neutrophils, has emerged as a promising frontier in cancer immunotherapy.^1^ Neutrophils, which arise from the myeloid hematopoietic lineage, constitute the largest proportion of circulating leukocytes in humans.^2^ As rapid first responders to pathogen invasion and tissue injury, they play indispensable roles in inflammation and host defense. Historically, neutrophils have been underestimated in cancer biology, largely due to their short lifespan and rapid turnover in peripheral blood. However, growing evidence now underscores their pivotal role as regulators of tumor growth and metastasis.^3^ In cancer patients, circulating neutrophil numbers frequently increase, and an elevated neutrophil-to-lymphocyte ratio has been documented as a poor prognostic marker across various malignancies.^4^ Moreover, high peripheral neutrophil counts are often accompanied by increased infiltration of neutrophils into solid tumors.^5,6^ While excessive neutrophil accumulation is generally linked to tumor progression and worse prognosis,^7,8^ we and others have demonstrated that neutrophils can also exert potent tumor-suppressive effects, particularly during early stages of tumor development.^9–11^ Notably, several recent findings indicate that successful T cell-based immunotherapies may rely on a coordinated neutrophil response to achieve robust anti-tumor effects.^12,13^ These observations collectively suggest that neutrophils undergo a dynamic and context-dependent phenotypic shift during cancer progression, transitioning from anti-tumoral to pro-tumoral state.

To better define this functional heterogeneity, a classification system analogous to that of tumor-associated macrophages (TAMs) has been proposed for tumor-associated neutrophils (TANs). Just as macrophages are broadly categorized as M1 (anti-tumoral) or M2 (pro-tumoral), TANs can be skewed toward an N1 (anti-tumoral) or N2 (pro-tumoral) phenotype depending on local signals in the tumor microenvironment.^13,14^ Type I interferons (IFNs) have emerged as key drivers of N1 polarization, conferring pro-inflammatory and anti-tumorigenic properties.^14^ In contrast, transforming growth factor beta (TGF-β) has been shown to promote the alternative N2 phenotype, characterized by immunosuppressive functions and support of tumor progression.^14^ Importantly, preclinical evidence suggests that neutrophils, if appropriately reprogrammed, could become powerful allies in cancer immunotherapy.^15,16^ For instance, inhibition of TGF-β signaling—using receptor kinase inhibitors such as SM16 or neutralizing antibodies—has been demonstrated to increase the recruitment of TANs with enhanced cytotoxicity and higher expression of pro-inflammatory cytokines.^16^ This shift toward an N1-like state is associated with reduced tumor growth and improved activation of cytotoxic CD8⁺ T cells. Notably, depletion of neutrophils in these models abolishes the therapeutic benefits of TGF-β blockade, underscoring the indispensable role of neutrophils in mediating anti-tumor effects under specific conditions.

Consistent with these findings, we and others have shown that type I IFNs promote TAN polarization toward the N1 phenotype, thereby enhancing their tumoricidal functions.^14,15,17–23^ This polarization is characterized by upregulation of immune-stimulatory molecules, increased production of reactive oxygen species, and enhanced antigen presentation capacity. TANs activated by type I IFNs exhibit greater cytotoxicity toward tumor cells, further highlighting their potential in orchestrating effective anti-tumor immunity. Moreover, type I IFNs modulate neutrophil lifespan and migration, facilitating their retention and activity within the tumor microenvironment (TME). In contrast, loss of type I IFN signaling—such as IFN-β deficiency- leads to the emergence of pro-tumoral neutrophils with impaired tumor cell killing, reduced activation marker expression, and prolonged survival that ultimately favors tumor progression and metastasis. These insights underscore the prospect of therapeutic strategies that aim to reprogram neutrophils by targeting deleterious signaling pathways rather than eliminating them or imparing their migration for establishing a successful therapies. Such approaches could complement existing immunotherapies by tipping the balance toward an anti-tumoral neutrophil phenotype within the TME.

Intriguingly, our previous work and that of others have identified signal transducer and activator of transcription 3 (STAT3) as a key factor driving the pro-tumoral (N2) phenotype in neutrophils with deficient type I IFN signaling.^11,17^ STAT3 is a well-established oncogenic transcription factor that is aberrantly activated in many human cancers, where it drives tumor cell proliferation, survival, angiogenesis, and immune evasion.^24^ Beyond its intrinsic tumor cell effects, STAT3 signaling plays a critical role in shaping the immune microenvironment. In immune cells, such as neutrophils, macrophages, dendritic cells, and regulatory T cells, persistent STAT3 activation promotes immunosuppression by upregulating molecules such as PD-L1 that dampen cytotoxic T cell responses.^11,24^ Despite extensive research into oncogenic roles of STAT3 signaling, its specific function within neutrophils has remained underexplored. Here, we demonstrated that STAT3 expression and activation are markedly elevated in pro-tumoral TANs in cancer patients. We further showed that neutrophil-specific STAT3 deletion enhances their anti-tumoral properties, stimulates cytotoxic CD8⁺ T cell responses that in turn leads to reduced tumor burden. Furthermore, therapeutic targeting of STAT3 in neutrophils, both in vitro and in vivo, amplified their capacity to activate cytotoxic T cells and promote effective tumor cell killing. Together, our findings highlight the potential of neutrophil-specific STAT3 inhibition as a promising strategy for harnessing myeloid cells in cancer immunotherapy and provide new insights into reprogramming the innate immune compartment to strengthen anti-tumor immunity.

## Results

### Elevated STAT3 expression in tumor-associated neutrophils is correlated with patient outcome

**Fig. 1:**
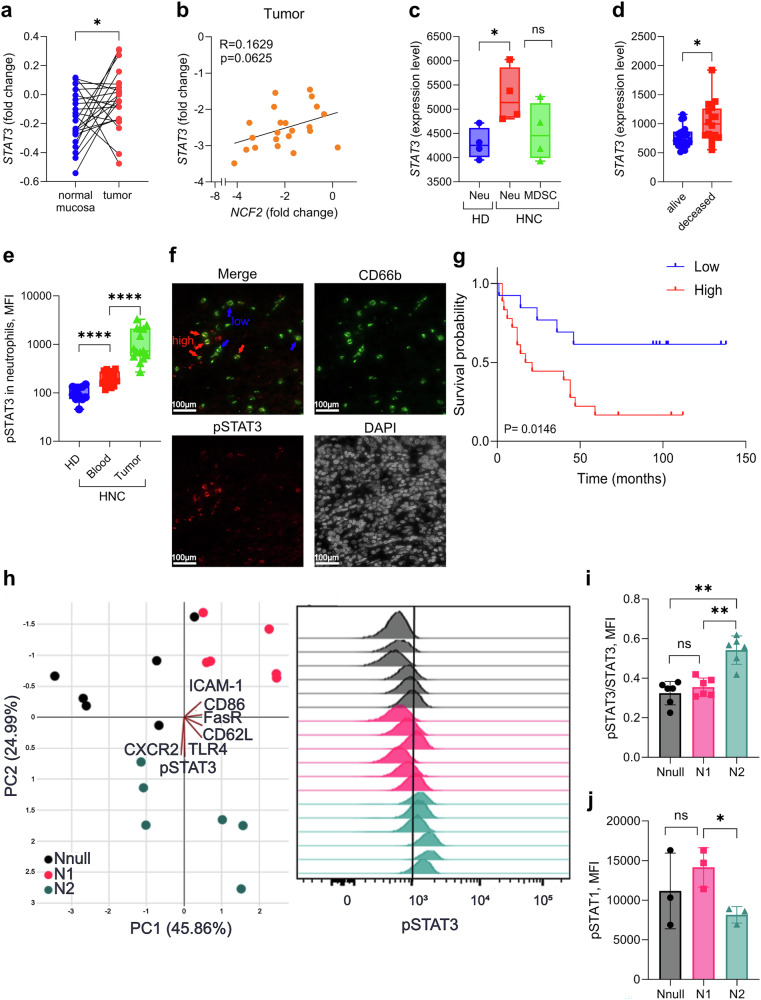
Pro-tumoral neutrophils show hyperactivated STAT3 signaling and contribute to poor disease outcomes in cancer patients. **a** Analysis of transcriptomic data from the GEO dataset GSE83519, showing the fold changes in STAT3 mRNA expression between normal mucosa and matching tumor tissue from head and neck cancer (HNC) patients. **b** Correlation analysis of the transcription levels (fold changes) of *STAT3* and neutrophil cytosolic factor 2 (*NCF2*) in tumor tissues. Data were analyzed from the GEO dataset GSE83519. **c** Analysis of transcriptomic data from the GEO dataset GSE79404, showing the fold change in STAT3 mRNA expression between blood neutrophils (PMNs) from healthy donors (HDs) (*blue*) and blood neutrophils (PMNs) (*red*) and myeloid-derived suppressor cells (MDSCs) (*green*) from HNC patients. **d** Analysis of transcriptomic data from the GEO dataset GSE122272, showing the fold changes in STAT3 mRNA expression in HNC patients with a 3-year overall survival status. Disease outcome: alive (*blue*) and deceased (*red*). **e** Flow cytometry analysis comparing pSTAT3 levels in blood neutrophils from HDs (*blue*) with those in blood (*red*) and tumor-associated neutrophils (TANs) (*green*) from HNC patients. **f** Representative images of immunofluorescence of tumor tissues from HNC patients showing high (*red arrow*) and low (*blue arrow*) pSTAT3 expression in TANs; scale bars, 100 μm. **g** Survival curves of HNC patients with a 5-year overall survival status showing high (*red*, *n* = 20) and low (*blue*, *n* = 13) pSTAT3 expression in TANs. **h**–**k** Neutrophils were isolated from the peripheral blood of healthy donors and polarized into N1 and N2 neutrophils, and some cells were left unpolarized (Nnull). **h** Left panel, principal component analysis showing the expression of ICAM-1, CD86, FasR, CD62L, TLR4, CXCR2 and pSTAT3 in healthy blood neutrophils in vitro polarized into pro- (N2) (*blue*) and antitumoral (N1) (*pink*) states and left unpolarized (Nnull) (*black*). Right panel, histograms of pSTAT3 in Nnull (*black*), N2 (*blue*) and N1 (*pink*) neutrophils. **i** Bar graphs showing the phosphorylation level of STAT3 in Nnull (*black*), N2 (*blue*) and N1 (*pink*) neutrophils as MFI of the pSTAT3/STAT3 ratio. **j** Bar graphs showing the MFI of pSTAT1 in Nnull (*black*), N2 (*blue*) and N1 (*pink*) neutrophils. Statistical analyses were performed via the Mann‒Whitney test. For the correlation analysis, the statistical significance and coefficient of determination were calculated using a Pearson correlation test, the Kaplan–Meier method was used to analyze tumor survival data, and the log-rank test was used for univariate analyses to compare survival curves among different groups. *P < 0.05, **P < 0.01, ***P < 0.001, ****P < 0.0001

Accumulating evidence confirms the pivotal role of neutrophils in cancer development and progression. As a result, numerous studies are ongoing to identify factors that drive the pro- or antitumoral bias of neutrophils. Previously, we reported that STAT3 is expressed in mouse protumoral neutrophils.^11,17^ To validate STAT3 expression in cancer patients, we first analyzed publicly available datasets of head and neck cancer (HNC) (GSE83519, GSE79404, and GSE122272) and melanoma (GSE114445) samples. Indeed, we discovered significantly elevated expression of *STAT3* in tumors compared with paired normal mucosa tissue (for HNC) and normal skin (for melanoma) (Fig. 1a and Supplementary Fig. 1a, respectively). Interestingly, we observed that increased *STAT3* expression in tumor tissue was positively correlated with the expression of neutrophil-specific markers in both cancer types (NCF2 for HNC and CD66b, MPO, and NCF1 for melanoma), suggesting that neutrophils play a role in the high expression of *STAT3* in tumors (Fig. 1b and Supplementary Fig. 1b–d). Further analyses (GSE79404) revealed significantly elevated *STAT3* expression in the blood neutrophils (polymorphonuclear neutrophils, PMNs) of HNC patients compared with healthy donor (HD) blood neutrophils (Fig. 1c). This dataset also revealed elevated *STAT3* expression in blood neutrophils compared with that in blood myeloid-derived suppressor cells (MDSCs) from the same patient (Fig. 1c). We also discovered that high *STAT3* expression (GSE122272) in tumors significantly correlated with poor patient outcomes (Fig. 1d).

To verify STAT3 activity in neutrophils at the protein level, we evaluated tyrosine-phosphorylated STAT3 (pSTAT3) levels in neutrophils from the tumor tissue and blood of HNC patients via flow cytometry. Compared with blood neutrophils from HDs, blood and tumor-associated neutrophils from cancer patients presented significantly higher expression of pSTAT3 (Fig. 1e). Interestingly, within the high pSTAT3-expressing TANs of HNC patients, we identified two subgroups with high and low pSTAT3 signatures (Fig. 1e). Next, with immunofluorescence analysis of tumor tissues from HNC patients, we confirmed the presence of TANs with high or low pSTAT3 expression (Fig. 1f). Notably, we discovered that patients with high pSTAT3 levels in TANs had worse outcomes (Fig. 1g).

Next, to assess the association of STAT3 signaling with the protumoral bias of neutrophils, we isolated blood neutrophils from healthy donors and primed them in vitro into the N1 or N2 phenotype (Supplementary Fig. 1e, for the gating strategy), as described previously.^25^ In brief, N1 neutrophils are primed with IFN-β, IFN-γ and LPS, whereas N2 neutrophils are primed with TGF-β, L-lactate, adenosine, IL-10, prostaglandin E2, and G-CSF. Neutrophil polarization was assessed by the expression of ICAM-1, CD86, FasR (for N1 neutrophils) or CXCR2, VEGF and CD62L (for N2 neutrophils) (Fig. 1h and Supplementary Fig. 1f). Consistent with the protumoral role of STAT3, we detected significantly high pSTAT3 levels in neutrophils with a protumoral (N2) phenotype (Fig. 1h). Moreover, N2 neutrophils also displayed the highest phosphorylation rate of STAT3 compared to N1 and unstimulated blood neutrophils (Nnull) (Fig. 1i). In contrast, we detected higher expression of antitumoral pSTAT1 protein in N1 neutrophils than in both Nnull and N2 neutrophils (Fig. 1j). Taken together, these results confirm that STAT3 signaling is highly activated in protumoral neutrophils and might play a role in cancer progression.

### Neutrophil-specific deletion of *STAT3* impairs tumor growth and progression

To understand the involvement of STAT3 signaling in tumor-related neutrophil functions in vivo, we generated a neutrophil-specific *STAT3* knockout mouse strain (*Ly6G*^*cre*^*STAT3*^*fl/fl*^, referred to as NStat3^−/−^). This model effectively carries out *STAT3* deletion restricted to Ly6G^+^ neutrophils (Supplementary Figs. 2a–d (for the gating strategy) and 3a–c). We demonstrated that STAT3 deficiency in neutrophils elevated the cytotoxicity of CD8^+^ T cells in naive mice (Supplementary Fig. 3d, e). Analysis of basic neutrophil functions revealed that the functionality of neutrophils from naive NStat3^−/−^ mice is comparable to that of neutrophils from naive *Ly6G*^*wt*^*STAT3*^*fl/fl*^ mice (these mice are referred to as wild type (WT) throughout this paper), except for a slightly altered CXCR2^+^CXCR4^−^ profile (Supplementary Fig. 3f–h).

Neutrophils have been reported to induce tumor growth and metastasis via the support of angiogenesis or neutrophil extracellular trap (NET) formation.^2,8,17,20,21,26–30^ Therefore, we isolated neutrophils from the bone marrow of NStat3^−/−^ and WT mice and measured their angiogenic potential and NET-forming capacity (after phorbol-12-myristat-13-acetate (PMA) stimulation). Compared with their WT counterparts, neutrophils with disturbed STAT3 signaling had reduced angiogenic activity and did not promote new vessel formation and sprouting (Supplementary Fig. 3i). Moreover, these neutrophils also had a reduced capacity to form NETs (Supplementary Fig. 3j).

**Fig. 2:**
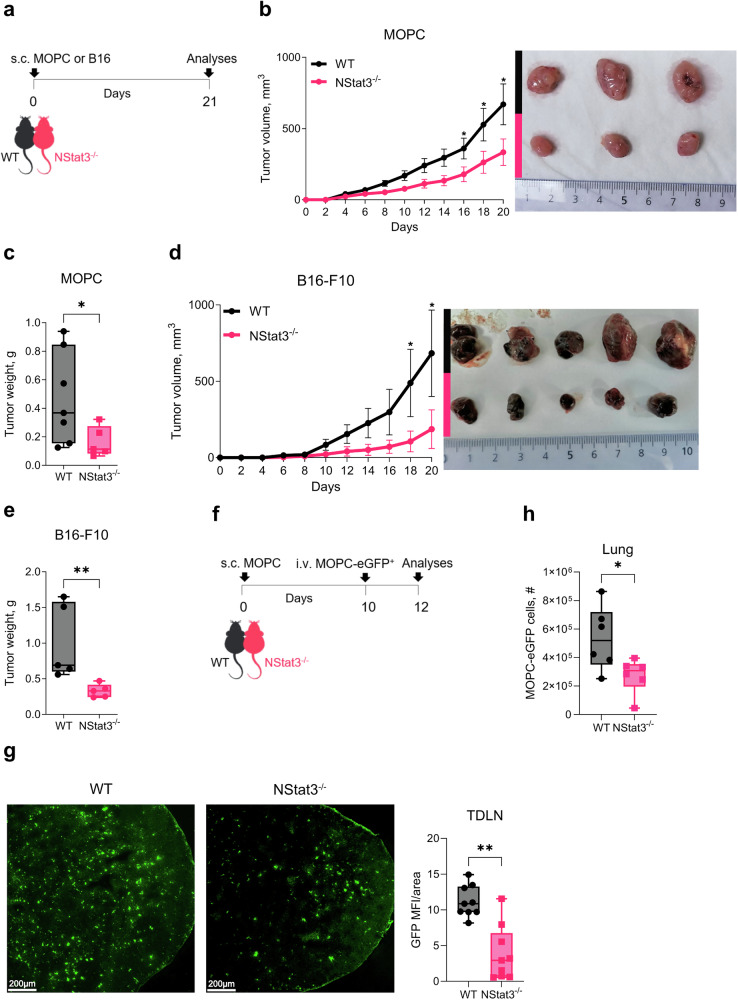
Targeting STAT3 signaling in a neutrophil-specific manner significantly impaired tumor progression in mice. **a** Schematic representation of the experimental tumor model used for analyses. **b** Tumor growth curves showing the average tumor volume in WT (*black*) and NStat3^−/−^ (*pink*) mice after MOPC injection (*left panel*). Representative image of tumors measured on day 21 (*right panel*). The error bars represent the means ± SEMs. The data are representative of three independent experiments (*n* = 8–10 mice per group). **c** Bar graph of tumor weights after 21 days in WT (*black*) and NStat3^−/−^ (*pink*) mice injected with MOPC. **d** Tumor growth curves showing the average tumor volume in WT (*black*) and NStat3^−/−^ (*pink*) mice after B16-F10 injection (*left panel*). Representative image of tumors measured on day 21 (*right panel*). The error bars represent the means ± SEMs. The data are representative of one experiment (*n* = 5 mice per group). **e** Bar graph of tumor weights after 21 days in WT (*black*) and NStat3^−/−^ (*pink*) mice injected with B16-F10 cells. **f** Schematic representation of the experimental tumor model used for metastasis analyses. **g** Representative IF images of TDLNs from WT and NStat3^−/−^ mice showing the presence of MOPC-eGFP^+^ tumor cells (*left panel*). Bar graph of the GFP signal/area in TDLNs of WT (*black*) and NStat3^−/−^ (*pink*) mice measured by FIJI (ImageJ) software (*right panel*). **h** Bar graph of the number of MOPC-eGFP^+^ tumor cells in the lungs of WT (*black*) and NStat3^−/−^ (*pink*) mice, as measured by flow cytometry. Statistical analyses were performed using Mann-Whitney-Test. *P < 0.05, **P < 0.01, ***P < 0.001, ****P < 0.0001

Next, we evaluated the effect of impaired neutrophil-STAT3 signaling on tumor growth and progression. For that purpose, NStat3^−/−^ and WT mice were subcutaneously engrafted with mouse oropharyngeal cancer cells (MOPCs) as described previously^11^ or with B16-F10 melanoma cells (Fig. 2a). Importantly, we observed that neutrophil-specific STAT3 knockout significantly impaired tumor growth in both cancer models (Fig. 2b–e), suggesting its pancancer effect.

To investigate tumor cell spread in NStat3^−/−^ mice, we used an experimental metastasis model that we established previously^18^ in which MOPC-eGFP^+^ tumor cells were intravenously injected into tumor-bearing mice (Fig. 2f). Microscopic analysis of tumor-draining lymph nodes (TDLNs) revealed fewer metastatic seeding in NStat3^−/−^ mice than in their WT counterparts (Fig. 2g). Reduced metastatic seeding in the lungs of NStat3^−/−^ mice was also confirmed by reduced numbers of MOPC-eGFP^+^ cancer cells detected by flow cytometry (Fig. 2h).

Neutrophils have been suggested to support metastasis into distant organs by escorting tumor cells that are protected inside NETs.^31,32^ To assess this, we measured the NET content in the blood of tumor-bearing WT and NStat3^−/−^ mice and found significantly less NET formation in neutrophils from tumor-bearing NStat3^−/−^ mice, compared to WT (Supplementary Fig. 3k). This finding was in line with the lower number of metastases in these mice.

Collectively, these results show that targeting STAT3 signaling specifically in neutrophils significantly impairs tumor progression in mice.

### STAT3-deficient neutrophils exhibit an antitumoral signature

**Fig. 3:**
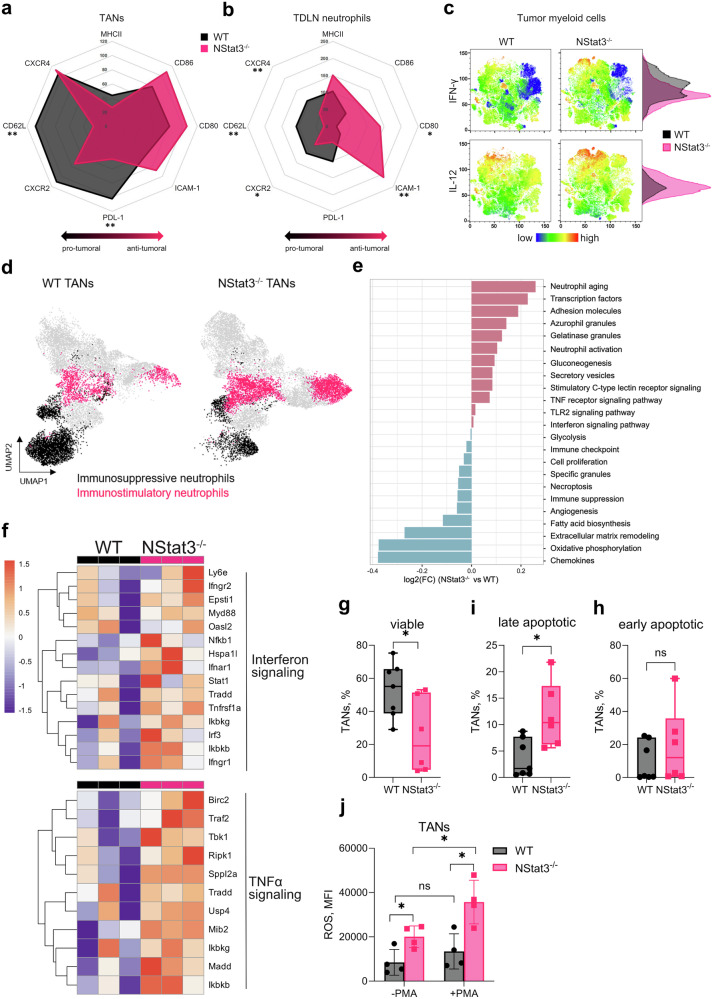
STAT3-deficient neutrophils obtain an antitumoral phenotype with increased expression of T cell stimulatory molecules. WT and NStat3^−/−^ mice were injected with MOPC as described in Fig. 2a. Spider web charts showing the expression of the indicated markers as MFI measured by flow cytometry on **a** TANs and **b** TDLN neutrophils from WT (*black*) and NStat3^−/−^ (*pink*) mice. **c** t-SNE plots with adjacent histograms showing the expression of IFN-γ and IL-12 measured by high-parameter single-cell flow cytometry in myeloid cells isolated from tumors of WT (*black*) and NStat3^−/−^ (*pink*) mice. **d** UMAPs of TANs isolated from WT and NStat3^−/−^ mice. The colors indicate the IFN-γ^lo^PDL-1^hi^MHCII^lo^CD80^lo^CXCR2^hi^CD62L^hi^ICAM1^lo^ (*black*) and IFN-γ^hi^PDL-1^lo^MHCII^hi^CD80^hi^CXCR2^lo^CD62L^lo^ICAM1^hi^ (*pink*) populations obtained by high-parameter single-cell flow cytometry. **e** Gene Ontology (GO) term analysis of up- or downregulated pathways in the TANs of NStat3^−/−^ mice compared with those of WT mice (bulk RNA-seq; *n* = 3 mice/condition). **f** Heatmaps showing altered expression of genes related to interferon and TNFα signaling from bulk RNA-seq of TANs from WT (*black*) and NStat3^−/−^ (*pink*) mice. The color scale indicates the log2-fold change in transcripts per million (TPM) for each gene, calculated as the ratio to the average TPM of control samples (*n* = 3 biological replicates). **g**–**i** Bar graphs of **g** viable (Annexin V and 7-AAD double negative), **h** early apoptotic (7-AAD negative, PE Annexin V positive) and **i** late apoptotic (Annexin V and 7-AAD double positive) TANs from WT (*black*) and NStat3^−/−^ (*pink*) mice as percentages detected by the Annexin V apoptosis assay. **j** Bar graph of ROS production in TANs from WT (*black*) and NStat3^−/−^ (*pink*) mice as a percentage, as measured by flow cytometry. The data are representative of three independent experiments (*n* = 5–7 mice per group). Statistical analyses were performed using Mann-Whitney-Test. *P < 0.05, **P < 0.01, ***P < 0.001, ****P < 0.0001

Disruption of STAT3 signaling in neutrophils impairs cancer development; however, the mechanism involved in this phenomenon is not clear. Therefore, we next examined and compared the phenotypes of neutrophils isolated from the tumors and TDLNs of WT and NStat3^−/−^ mice. Compared with their WT counterparts, STAT3-deficient TANs exhibit a significant signature of antitumor biomarkers on their surface (Fig. 3a). Moreover, this phenotypical divergence of neutrophils was even more evident in TDLNs (Fig. 3b). TANs from NStat3^−/−^ mice presented significantly elevated expression of CD80, CD86 and ICAM-1, together with reduced levels of CD62L and PD-L1 (Fig. 3a). Notably, PD-L1 is a key mediator of tumor immune suppression and a primary target of current immunotherapies through checkpoint inhibition. TDLN neutrophils further increase the expression of molecules involved in the activation of adaptive immune responses (MHC II, CD80, CD86 and ICAM-1) (Fig. 3b). Similar phenotypical changes in STAT3-deficient TANs and TDLN neutrophils were also observed in the B16-F10 tumor model (Supplementary Fig. 4a, b).

To gain deeper insight into the phenotypical alteration of STAT3-deficient neutrophils, we performed high-parameter single-cell flow cytometry analysis and observed significant changes in the myeloid cell landscape of the tumor (Supplementary Fig. 4c). Moreover, deletion of STAT3 resulted in increased IFN-γ and IL-12 expression in tumor myeloid cells (Fig. 3c). Disruption of neutrophil-derived STAT3 resulted in changes in the entire interdependent myeloid cell landscape in the TME. Nonetheless, the most significant changes were observed in the neutrophil compartment (Fig. 3d). Enrichment of antitumor neutrophil populations with elevated IFN-γ, ICAM-1, MHCII, CD80 and iNOS signatures in tumors from NStat3^−/−^ mice were observed, as compared to WT tumors (Fig. 3d). In contrast, in WT tumors protumoral populations with elevated CXCR2, CD62L and PD-L1 were enriched (Fig. 3d).

To explore the mechanism underlying the upregulation of ICAM-1, CD80, CD86, and MHCII in STAT3-deficient neutrophils, we performed bulk RNA sequencing of TANs isolated from WT and NStat3^−/−^ mice. Transcriptomic analysis revealed strong upregulation of pathways related to interferon (IFN) signaling, TNFα signaling and adhesion molecules in NStat3^−/−^ TANs, whereas tumor-promoting pathways, e.g., extracellular matrix remodeling, angiogenesis, immune checkpoint and immune suppression, were downregulated (Fig. 3e). These pathways are closely linked to the phenotypic and functional changes we observed in STAT3-deficient neutrophils. For instance, interferon signaling is known to induce the expression of MHCII molecules and costimulatory molecules such as CD80 and CD86 on myeloid cells, thereby enhancing their antigen-presenting capacity.^22^ We observed that many genes involved in interferon signaling are elevated in TANs isolated from NStat3^−/−^ mice (Fig. 3f). TNFα signaling promotes the expression of ICAM-1, which facilitates immune cell adhesion and supports neutrophil–T cell interactions. Our bulk RNA sequencing data also revealed that NStat3^−/−^ TANs presented increased expression of genes involved in TNFα signaling (Fig. 3f). Furthermore, we discovered that many genes related to phagocytosis, endosome-to-lysosome transport together with antigen presentation and adhesion molecules are upregulated in TANs isolated from NStat3^−/−^ mice compared with their WT counterparts (Supplementary Fig. 4d–f). The upregulation of phagocytosis-related genes suggests increased tumor antigen uptake, which, together with increased endosome-to-lysosome transport and antigen presentation pathways, likely contributes to increased MHCII surface expression and overall improved immunostimulatory function. Importantly, we also found that CD8^+^ T cells are more abundant and in close contact with low pSTAT3-expressing neutrophils in tumors from HNC patients (Supplementary Fig. 4g).

STAT3 signaling is an important pathway involved in cell survival.^24^ In agreement, elevated survival and reduced apoptosis are hallmarks of pro-tumoral neutrophils.^19^ We assessed the survival of neutrophils isolated from NStat3^−/−^ mice and observed that these cells were less viable and showed increased apoptosis (Fig. 3g–i). Moreover, we compared the reactive oxygen species (ROS) response in TANs from WT and NStat3^−/−^ mice and found that neutrophils from NStat3^−/−^ mice produced significantly higher amounts of ROS than their WT counterparts did after PMA stimulation (Fig. 3j).

These data show that STAT3-deficient neutrophils acquire an antitumoral phenotype with elevated apoptosis and increased expression of T cell stimulatory molecules.

### STAT3-deficient neutrophils elicit antitumor cytotoxic T cell responses

**Fig. 4:**
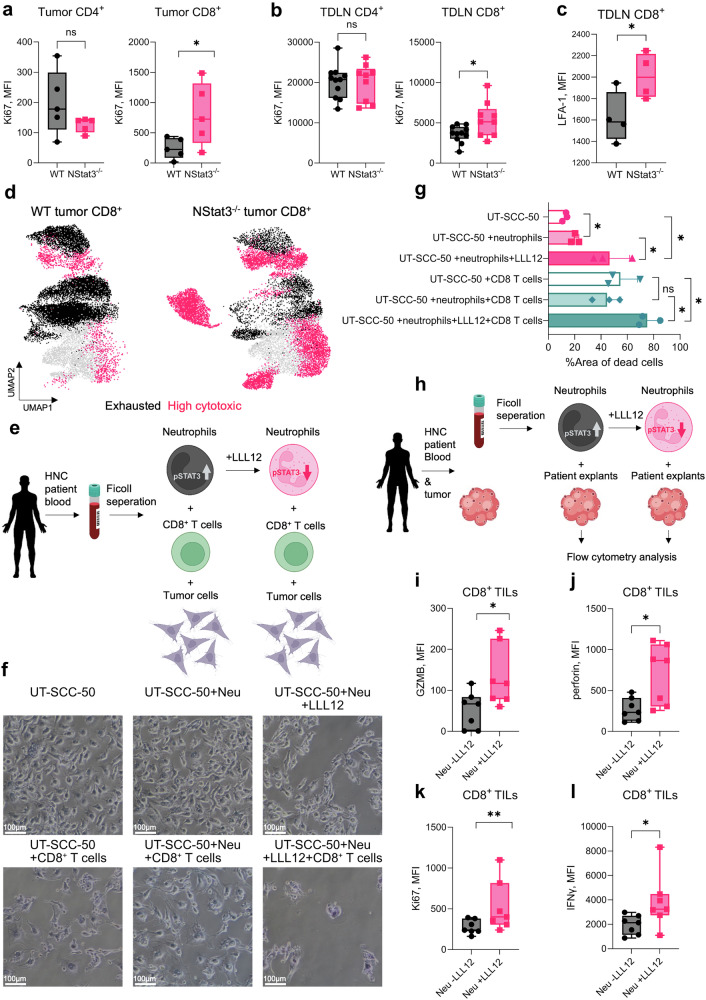
Targeting STAT3 in neutrophils enhances their capacity to stimulate an effective anticancer CD8^+^ T cell response. WT and NStat3^−/−^ mice were injected with MOPC as described in Fig. 2a (*n* = 5 mice per group). Bar graphs showing the expression of Ki67 in CD4^+^ (*left panel*) and CD8^+^ (*right panel*) T cells isolated from **a** tumors and **b** TDLNs of WT (*black*) and NStat3^−/−^ (*pink*) mice. **c** Bar graph showing the expression of LFA-1 in CD8^+^ T cells isolated from TDLNs of WT (*black*) and NStat3^−/−^ (*pink*) mice. **d** UMAP plots of tumor-infiltrating CD8^+^ T cells isolated from WT (*left panel*) and NStat3^−/−^ (*right panel*) mice. The colors indicate the IFN-γ^mid^Ki67^lo^PD-1^hi^LAG-3^hi^GZMB^lo^CTLA-4^hi^TIGIT^hi^perforin^lo^ (*black*) and IFN-γ^hi^Ki67^hi^PD-1^lo^LAG-3^lo^GZMB^hi^CTLA-4^mid^TIGIT^lo^perforin^hi^ (*pink*) populations obtained by high-parameter single-cell flow cytometry. **e** Schematic explanation of the experimental design for Fig. 4f. Briefly, neutrophils and CD8^+^ T cells were isolated from the blood of HNC patients. Neutrophils were treated with ±LLL12 and cocultured with CD8^+^ T cells and later with tumor cells. **f** Microscopy images of UT-SCC-50 cells cocultured with the indicated immune cells and ±LLL12. The details are described in the Materials & Methods and the tumor killing assay section. **g** Bar graph of microscope images; the percentage of the empty area was quantified by FIJI (ImageJ) software. **h** Schematic explanation of the experimental design for (**i**–**l**). In brief, neutrophils were isolated from the blood of HNC patients, treated with ±LLL12, and coincubated with tumor explants obtained from the same patient. **i**–**l** Bar graphs showing the expression of **i** granzyme B, **j** perforin, **k** Ki67 and **l** IFN-γ in CD8^+^ T cells isolated from patient tumor explants coincubated with neutrophils treated with (*pink*) or without (*black*) LLL12. The data are representative of three independent experiments. Statistical analyses were performed using Mann-Whitney-Test. *P < 0.05, **P < 0.01, ***P < 0.001, ****P < 0.0001

Given that surface molecules and protein mediators involved in T cell proliferation and activation are upregulated in STAT3-deficient neutrophils, we next assessed whether these cells can effectively modulate T cell responses in tumor-bearing mice. To this end, we first assessed the proliferation of T cells (Ki67) in tumors and TDLNs from NStat3^−/−^ and WT mice via flow cytometry. Interestingly, we found that the proliferation of CD8^+^ T cells, but not that of CD4^+^ T cells, was significantly elevated in both tumors and TDLNs from NStat3^−/−^ mice (Fig. 4a, b, respectively). Moreover, CD8^+^ T cells from NStat3^−/−^ mice presented increased expression of LFA-1, the binding partner of ICAM-1, which is essential for efficient T cell stimulation (Fig. 4c).

Next, we assessed changes in the CD8^+^ T cell compartment in tumor-bearing NStat3^−/−^ mice. High-parameter cell analysis of tumors and TDLNs revealed significant enrichment of the cytotoxic IFN-γ^hi^Ki67^hi^granzyme B (GZMB)^hi^perforin^hi^ population of CD8^+^ T cells in NStat3^−/−^ mice, whereas in WT mice, the noncytotoxic T cell population with the IFN-γ^lo^Ki67^lo^GZMB^lo^perforin^lo^ signature was predominant (Fig. 4d and Supplementary Fig. 5a).

To mechanistically validate the role of STAT3 in stimulating cytotoxic CD8^+^ T cell responses in human neutrophils, we blocked STAT3 phosphorylation in neutrophils isolated from HNC patient blood via the small molecule inhibitor LLL12 (Supplementary Fig. 5b) and stimulated autologous CD8^+^ T cells with such neutrophils (LLL12 was removed after 1 h of neutrophil stimulation) (Fig. 4e). We observed that targeting STAT3 phosphorylation in human neutrophils stimulated antitumoral N1 bias (Supplementary Fig. 5c–h), which was comparable to that in mouse neutrophils. Moreover, compared with CD8^+^ T cells stimulated with untreated neutrophils, patient CD8^+^ T cells educated with LLL12-treated neutrophils presented a significantly greater killing capacity toward human laryngeal squamous cell carcinoma UT-SCC-50 tumor cells (Fig. 4e–g).

Next, we tested the antitumoral potential of patient LLL12-treated neutrophils via three-dimensional (3D) patient-derived tumor explants (HNC, experimental schema Fig. 4h). In agreement with the in vivo results from the mice, we observed significant expansion and activation of cytotoxic GZMB^hi^ perforin^hi^ Ki67^hi^ IFN-γ^hi^ cytotoxic CD8^+^ T cells in these explants but not in those incubated with STAT3-sufficient neutrophils (Fig. 4i–l).

Finally, to validate the involvement of CD8^+^ T cells in the antitumor effects observed in NStat3^−/−^ mice, we depleted CD8^+^ T cells in MOPC-injected WT and NStat3^−/−^ mice (Supplementary Fig. 5i). Our results showed that CD8^+^ T cell depletion completely abrogated the tumor growth inhibition observed in NStat3^−/−^ mice. In fact, tumor growth in CD8-depleted NStat3^−/−^ mice was restored to levels comparable to those in WT controls (Supplementary Fig. 5j). These findings confirm our hypothesis that the antitumor effects of STAT3-deficient neutrophils are dependent on the presence and functionality of CD8^+^ cytotoxic T cells.

Together, these data demonstrate that targeting neutrophil-STAT3 significantly enhances their capacity to elicit antitumoral cytotoxic T cell responses. This effect is observed not only in experimental mouse cancer models but also in the human system.

### Therapeutic targeting of Stat3 in tumor-associated neutrophils via intratumoral CpG-Stat3ASO injection shows great potential for cancer treatment

**Fig. 5:**
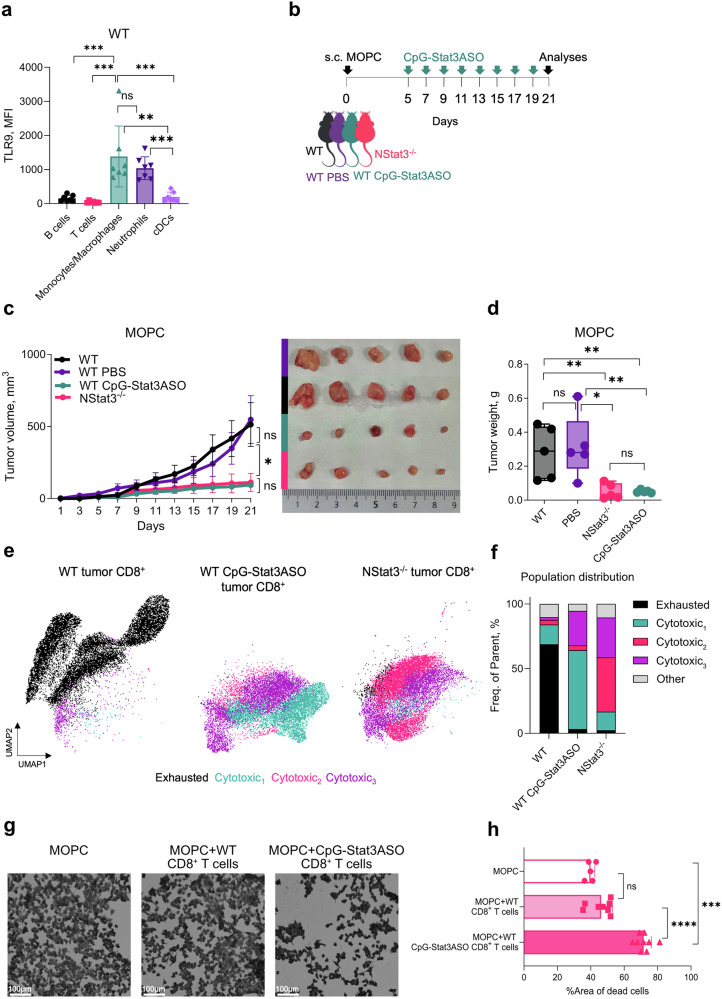
Targeting STAT3 in TANs with CpG-Stat3ASO leads to the enrichment of effector CD8^+^ T cells in tumors. **a** Bar graph showing the expression of TLR9 in immune cells in the tumor tissues of WT mice (*n* = 7 mice per group). **b** Schematic representation of the experimental tumor model used for intratumoral CpG-Stat3ASO injection experiments. **c** Tumor growth curves showing the average tumor volume in WT (*black*), intratumoral PBS-injected (*purple*), NStat3^−/−^ (*pink*) and intratumoral CpG-Stat3ASO-injected (*blue*) mice after MOPC injection (*left panel*). Representative image of tumors measured on day 21 (*right panel*). The error bars represent the means ± SEMs. (*n* = 5 mice per group). **d** Bar graph showing tumor weights on day 21 in WT (*black*), PBS-injected (*purple*), NStat3^−/−^ (*pink*) and CpG-Stat3ASO-injected (*blue*) mice. **e** UMAP plots and **f** stacked bar graphs of tumor-infiltrating CD8^+^ T cells isolated from WT (*left panel*), CpG-Stat3ASO (*middle panel*) and NStat3^−/−^ (*right panel*) mice. The colors indicate the IFN-γ^lo^Ki67^mid^PD-1^hi^LAG-3^hi^GZMB^lo^GZMK^mid^CTLA-4^hi^TIGIT^hi^perforin^lo^ (*black*), IFN-γ^hi^Ki67^mid^PD-1^lo^LAG-3^mid^GZMB^hi^GZMK^hi^CTLA-4^mid^TIGIT^mid^perforin^hi^ (*blue*), IFN-γ^mid^Ki67^hi^PD-1^lo^LAG-3^lo^GZMB^hi^GZMK^mid^CTLA-4^mid^TIGIT^mid^perforin^mid^ (*pink*) and IFN-γ^mid^Ki67^mid^PD-1^lo^LAG-3^mid^GZMB^hi^GZMK^hi^CTLA-4^lo^TIGIT^lo^perforin^mid^ (*purple*) populations obtained by high-parameter single-cell flow cytometry. **g** Microscopy images of MOPC cells cocultured with CD8^+^ T cells isolated from the tumors of WT and intratumorally CpG-Stat3ASO-injected mice. **h** Bar graph of the microscope images; the percentage of the empty area was quantified by FIJI (ImageJ) software. Statistical analyses were performed using Mann-Whitney-Test. *P < 0.05, **P < 0.01, ***P < 0.001, ****P < 0.0001

Given that the inhibition of STAT3 activity modulates neutrophil tumorigenic potential, we were next interested in whether the targeted silencing of STAT3 in neutrophils in vivo would suppress tumor growth. We considered that the administration of small-molecule STAT3 inhibitors could cause off-target effects in patients; thus, we decided to utilize myeloid cell- and neutrophil specific, antisense oligonucleotide technology to overcome this potential interference. To target TANs, we intratumorally injected CpG-Stat3ASO^33–35^, which targets TLR9-expressing neutrophils via a CpG oligodeoxynucleotide as a TLR9 ligand.^36,37^ Indeed, we confirmed that TANs from WT mice expressed significantly higher levels of TLR9 than other immune cells did, such as B cells, T cells and myeloid dendritic cells (Fig. 5a). We first tested the uptake of the CpGStat3ASO molecule by TANs with using a fluorescent reporter Cy3-CpGASO. These data showed that TANs effectively internalized CpG-ASO molecules as early as 30 min after incubation (Supplementary Fig. 6a).

After the intratumoral injection of CpG-Stat3ASO into WT mice (see the experimental scheme, Fig. 5b), we monitored tumor progression for 21 days. Importantly, we observed significantly reduced tumor growth and burden, which were comparable to those in NStat3^−/−^ mice (Fig. 5c, d). Moreover, high-parameter flow cytometry analysis of tumors and TDLNs revealed that the CD8^+^ T cell populations in the CpG-Stat3ASO-treated WT mice were phenotypically similar to those in the NStat3^−/−^ mice (Fig. 5e, f and Supplementary Fig. 6b, c). These CD8^+^ T cell populations highly expressed effector cytotoxic molecules, such as IFN-γ, GZMB, GZMK, and perforin, in contrast to those in WT mice, where nonfunctional, exhausted CD8^+^ T cell populations were enriched in tumors and TDLNs (Fig. 5e, f and Supplementary Fig. 6b, c). To exclude the possibility that TANs are activated solely by CpG molecules, we intratumorally injected MOPC-bearing mice with CpG versus CpG-Stat3ASO (Supplementary Fig. 6d) and showed that the arrested tumor growth together with enriched immunostimulatory TANs and highly cytotoxic CD8^+^ T cell populations in the TME were not influenced by CpG but were unique to CpG-Stat3ASO treatment (Supplementary Fig. 6e–g).

To further validate the elevated cytotoxicity of CD8^+^ T cells in CpG-Stat3ASO-injected mice, we isolated CD8^+^ T cells from tumors and incubated them with MOPC cells in vitro. After 24 h of incubation, compared with their WT counterparts, tumor-infiltrating CD8^+^ T cells from CpG-Stat3ASO-injected mice showed significantly increased killing capacity against MOPC cells (Fig. 5g, h).

Together, these data demonstrate that efficient knockdown of neutrophil STAT3 via the intratumoral injection of CpG-Stat3ASO supported the expansion of potently cytotoxic CD8^+^ T cells and therefore provides a promising strategy for cancer immunotherapy.

## Discussion

Neutrophils, the most abundant circulating leukocytes in humans, accumulate in solid tumors and significantly affect disease prognosis and outcome.^6^ Although the presence of neutrophils in tumors is often associated with poor prognosis in many cancer types,^38^ a growing body of evidence has demonstrated the essential role of these cells in successful cancer treatment. In fact, the antitumoral activities of neutrophils have been shown in many studies. In addition to direct killing of tumor cells, neutrophils employ a wide range of antitumor properties, one of which is the stimulation of antitumor T- or B-cell responses.^11,22,39^

Previously, we and others demonstrated that at the early stage of cancer, neutrophils initiate cytotoxic T cell responses via elevated expression of molecules involved in antigen presentation and T cell costimulation, not only at the tumor site but also in tumor-draining lymph nodes.^6,9,11,22^ However, these activities of neutrophils are inhibited by the immunosuppressive tumor microenvironment and by multiple cytokines or growth factors present in the tumor site.^11,40^ Research interest in identifying and targeting signaling pathways involved in the protumoral activation of neutrophils is currently growing. We and others have previously shown that the TGFβ and type I interferon signaling pathways govern the pro- and antitumoral functions of neutrophils, respectively. Our previous studies revealed that neutrophils with defective type I IFN signaling promote tumor growth and spread in mice. Interestingly, such neutrophils exhibit elevated STAT3 phosphorylation.^17^ The STAT3 signaling pathway can be activated by IL-6, G-CSF and other growth factors and governs multiple biological processes, such as proliferation, cell survival and differentiation of immune cells. Upon pathogenic invasion, STAT3 signaling regulates the emergency-granulopoiesis and induces a rapid increase in mature neutrophils in the circulation.^41^ However, this signaling pathway is hyperactivated in the tumor microenvironment of the majority of human cancers, leading to cancer cell survival, therapeutic resistance and immune evasion.^24^ The prolonged survival and persistence of neutrophils in tumors is another hallmark of protumoral neutrophils. Tumor-driven activation of STAT3 in neutrophils not only prolongs their survival in tumor tissues but also induces the expression of suppressive molecules for the T cell response, such as PD-L1.^11,40,42^ This adds another layer of complexity to the cellular sources of this key immune regulatory molecule.^43^ Since neutrophils are abundant in the tumor microenvironment and play roles in immune modulation, PD-L1 expression on neutrophils could significantly impact the effectiveness of checkpoint inhibitor therapies. Furthermore, in our study, we showed that neutrophil-derived STAT3 is upregulated in solid human tumors, such as HNC or melanoma, and it is correlated with poor disease outcomes. In line with these findings, healthy human neutrophils polarized into a protumoral (N2) state in vitro presented significant upregulation of STAT3.

To mechanistically assess the role of neutrophil-STAT3 in tumor growth and progression, we generated a mouse strain carrying neutrophil-specific knockout (NStat3^−/−^) by utilizing a Ly6G-Cre system, which has a high degree of specificity for neutrophils. We previously showed that there was no Cre activity in any analyzed hematopoietic stem or progenitor population, including GMPs, whereas transgene activity was immediately detectable upon neutrophil development. This explains the lack of Cre activity in peripheral leukocytes, which is distinct from that in neutrophils, and in a very small pool of eosinophils.^44^ Since the number of eosinophils within the tumor microenvironment is very low, compared with that in TANs, minor Cre activity in this population is negligible. Compared with WT mice, NStat3^−/−^ mice presented significantly impaired tumor growth and metastatic spread, in both HNC and melanoma models. The characterization of neutrophils from such tumor-bearing mice revealed their strong phenotypical bias toward antitumoral N1, with elevated expression of molecules involved in T cell activation. In a recent study, Wu et al. demonstrated that neutrophils exhibit antigen-presenting cell (APC) abilities comparable to those of professional APCs, such as dendritic cells, and evoke antitumor T cell responses.^6^ Interestingly, the same study also demonstrated that MHCII^+^ neutrophils show strong co-localization with CD8^+^ T cells and activate them in a nonspecific manner; mainly via ICAM-1/LFA-1 interaction. In line with these findings, we showed that STAT3-deficient neutrophils increase their expression of MHCII, CD80/86, and ICAM-1 and operate by licensing antitumoral cytotoxic CD8^+^ T cell responses rather than by direct killing of tumor cells. In the current literature, opinions on the influence of neutrophils on the antitumoral cytotoxic T cell response have not yet reached a consensus. Some studies have shown the negative effect of neutrophils on the CD8^+^ T cell response,^45,46^ whereas others have shown the necessity of neutrophils for the tumor cytotoxic activity of CD8^+^ T cells,^6,12,13^ once again revealing the functional heterogeneity of these cells in cancer. Here, we identified neutrophil-STAT3 as a negative modulator of the anticancer CD8^+^ T cell response. In neutrophil-STAT3 deficient mice, we observed a significant increase in CD8^+^ T cell populations with high cytotoxic signature, which is likely responsible for the observed decrease in tumor growth and metastasis.

Debate on the comparability between the mouse and human immune systems is ongoing. While some studies have shown that most neutrophil states identified in human tumors can also be found in mice,^47^ others have pointed on differences between these two systems.^48^ Here, we evaluated our findings in both mouse and human experimental settings. For example, targeting STAT3 phosphorylation in mouse neutrophils supported the ability of these cells to stimulate CD8^+^ T cells, the same phenomenon we observed in neutrophils derived from cancer patients. In agreement with these findings, enhanced antitumor activity of CD8^+^ tumor-infiltrating lymphocytes in patient 3D tumor explants was observed after stimulation with neutrophils with suppressed STAT3 signaling. These results emphasize conserved antitumoral neutrophil functions in both mice and humans.

The clinical translation of small-molecule STAT3 inhibitors faces challenges due to off-target effects on nonmalignant cells, such as the reduced antitumor activity of CD8^+^ T cells.^49^ To overcome this challenge, we utilized TLR9-specific antisense oligonucleotides recently developed for targeting myeloid suppressor cells in the microenvironment of solid tumors, such as prostate, renal or head and neck cancers.^33,35,50^ For targeted STAT3 downregulation in TANs, a STAT3 antisense molecule tagged with CpG (a TLR9 ligand) was generated. Given that mouse tumor macrophages, along with neutrophils, express TLR9, it is possible that such treatment could also impact macrophage activity. However, our data from a mouse transplantable tumor model indicate that targeting STAT3 only in neutrophils (NStat3^−/−^ mice) is sufficient to achieve a significant reduction in tumor growth. While neutrophils did not express STAT3 in our NStat3^−/−^ mice, we observed strong overexpression of this molecule in macrophages (possibly as a compensatory mechanism), and yet tumor growth was impaired in such mice. Targeting STAT3 in neutrophils and in macrophages leads to antitumor effects through different mechanisms. These findings suggest that STAT3 may play distinct roles in different myeloid cell populations. We demonstrated that the intratumoral injection of CpG-Stat3ASO into WT mice strongly impaired tumor growth and promoted the expansion of cytotoxic T cells, which was comparable to the results obtained in neutrophil-Stat3 knockout mice. These findings underscore the therapeutic potential of this treatment and identify neutrophils as the primary target of the treatment.

**Fig. 6:**
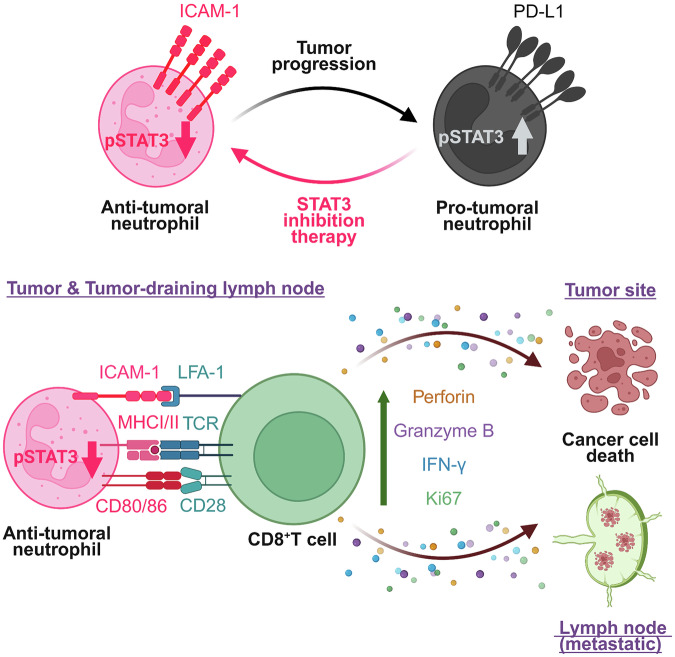
Graphical abstract. Created with BioRender.com

When activated accordingly, neutrophils perform antitumoral functions. Recent reports indicate that neutrophils are important not only for their antitumoral activity but also for licensing the antitumoral adaptive immune response. Consequently, neutrophils are emerging as essential cellular targets for cancer immunotherapy. However, the identification and precise targeting of specific pathways within neutrophils remain challenging. Our findings demonstrate that targeting STAT3 signaling in neutrophils presents a therapeutic opportunity to convert immunogenic “cold” tumors into a more immunogenic “hot” state, thereby enhancing cytotoxic T cell responses across different tumor types and suggesting that it could significantly improve the response to checkpoint therapy in vivo (Fig. 6). Taken together, our study shows that targeting solely neutrophil STAT3 with strategies such as CpG-STAT3ASO could provide an efficient and clinically relevant therapeutic strategy to maximize treatment efficacy in cancer.

## Materials and methods

### Human samples

The protocol was approved by the Ethics Committee of the University of Duisburg-Essen. All the subjects provided written informed consent in accordance with the Declaration of Helsinki.

A total of 66 patients (29 for flow cytometry, 34 for immunofluorescence and 3 for the tumor killing assay) (56 male and 10 female patients, with a mean age of 62 years (42–84)) with head and neck squamous cell carcinoma, who were scheduled for surgical resection, consented to tissue collection of a portion of the lymph nodes, tumor and/or blood for research purposes at the Otorhinolaryngology Department of the University Hospital Essen, Germany. Patients with other or unknown histological types of tumors, such as adenocarcinoma, sarcoma, lymphoma, cancer of unknown primary origin (CUP) and tumors associated with specific risk factors, such as nasal squamous cell carcinoma, which is affiliated with Epstein–Barr virus, as well as patients who received any cancer-associated therapies prior to the study were not included. Fresh LN tissue samples for clinical reasons were obtained after pathological examination, and all histological findings (the type of malignancy and metastatic status of the LN) were confirmed by experienced pathologists. The detailed characteristics of the patients are presented in Table S1.

### Human sample collection, processing and polarization of neutrophils

Fresh tumor samples were digested in an enzyme mixture (DMEMc (Dulbecco´s modified Eagle´s medium (Thermo Fisher Scientific, Massachusetts, USA) supplemented with 10% fetal bovine serum (PAN-Biotech, Aidenbach, Germany) and 100 U/mL penicillin‒streptomycin (Thermo Fisher Scientific)) supplemented with 0.2 μg/mL dispase, 0.2 μg/mL collagenase A, and 100 μg/mL DNase I (Merck, Darmstadt, Germany)), with 1 mL per sample for 45 min at 500 rpm and 37 °C. The cells were passed through 100 μm sterile strainers (Cell Trics, Partec, Sysmex Europe GmbH, Goerlitz, Germany) and centrifuged at 300 × *g* and 4 °C for 5 min, after which the supernatant was discarded. The pellet was resuspended in PBS (Dulbecco’s Phosphate Buffered Saline 1×, Thermo Fischer Scientific) containing Human BD Fc Block (1:500 concentration; BD Biosciences, Pharmingen, BD, New Jersey, USA). Clone: Fc1.3216, RRID:AB_2728082) and incubated for 10 min at 20 °C. Human peripheral blood was drawn into 3.2% sodium citrate anticoagulant monovettes (Sarstedt, Nümbrecht, Germany) and was collected within 2 h after collection.

For the polarization of healthy human neutrophils, peripheral blood was drawn into 3.2% sodium citrate anticoagulant monovettes (Sarstedt) and mixed 1:1 with PBS (Gibco, Thermo Fisher Scientific, Waltham, MA, USA) before separation by density gradient centrifugation (Biocoll density of 1,077 g/ml, Merck). The mononuclear cell fraction was discarded, and neutrophils were isolated by sedimentation over 1% polyvinyl alcohol, followed by hypotonic lysis (0.2% NaCl) of erythrocytes. Isolated neutrophils were plated into 24-well cell culture plates at 3 ml/well at a concentration of 5 × 10^6^/ml in complete medium supplemented with (MatTek Corporation, MA) either N1 or N2 polarization supplements. N1 polarization cocktail: 100 ng/ml lipopolysaccharide (Santa Cruz Biotechnology, Inc., Texas, USA), 50 ng/ml IFNγ (Gibco, PeproTech, Hamburg, Germany), and 10,000 U/ml IFN-β (Gibco, Peprotech) at 37 °C in a humidified air atmosphere containing 5% carbon dioxide (CO2). N2 polarization cocktail: 25 mM L-lactate (Merck), 10 μM adenosine (Merck), 20 ng/ml TGF-β (PeproTech), 10 ng/ml IL-10 (PeproTech), 20 ng/ml prostaglandin E2 (Merck), and 100 ng/ml granulocyte colony-stimulating factor (G-CSF; PeproTech) pH 6.7 in 5% CO2 at 37 °C. To increase the life span of neutrophils, all polarization media were supplemented with 3 μM of the pancaspase inhibitor QVD-Oph (Abcam, Cambridge, England). Control neutrophils (Nnull) were cultivated in complete medium containing 3 μM QVD-OPh. All polarization experiments were conducted at 37 °C in a humidified air atmosphere containing 5% CO2 for 24 h.

### Cell lines

The mouse oropharyngeal carcinoma cell line MOPC (C57BL/6-derived, HPV16 E6/E7^−^) was obtained as a gift from Dr. William Chad Spanos and John H. Lee (Sanford Research/University of South Dakota, Sioux Falls, SD, US) (PMID: 35833131, PMID: 19283850, PMID: 28300057). The cells were cultivated in special media (67% DMEM (Thermo Fischer Scientific), 22% Hams F12 nutrient mix (Thermo Fischer Scientific), 10% fetal bovine serum (PAN-Biotech), 1% penicillin‒streptomycin (Thermo Fischer Scientific), 0.5 µg/ml hydrocortisone (Merck), 8.4 ng/ml cholera toxin (Merck), 5 µg/ml transferrin (Merck), 5 µg/ml insulin (Merck), 1.36 ng/ml Tri-iodo-thyronine (Merck), and 5 µg/ml E.G.F. (Merck)). The melanoma B16-F10 tumor cell line was purchased from ATCC (CRL-6475). These cells were maintained in Iscove’s modified Dulbecco’s medium (IMDM) (Gibco) supplemented with 10% fetal bovine serum (PAN-Biotech), 1% penicillin‒streptomycin (Thermo Fischer Scientific), and 250 μmol/l β-mercaptoethanol (Gibco). The cells were grown in monolayers at 37 °C in a humidified CO_2_ incubator. Human laryngeal squamous cell carcinoma UT-SCC-50 (p53-frameshift, c.920del73/p14—No transcript/HPV16 E6/E7−) was obtained free of charge from University Hospital Ulm (PMID: 17312569, 18487078). The cells were cultivated in DMEMc supplemented with 1% nonessential amino acids (Gibco) and 1% L-glutamine (Gibco) freshly added to the cells. During cultivation, the cell lines were regularly tested for mycoplasma contamination, with negative results. The cells were grown in monolayers at 37 °C in a humidified incubator with 5% CO_2_. During cultivation, the cell lines were regularly tested for mycoplasma contamination, with negative results.

### Mouse study

For the transplantable mouse cancer model, we generated neutrophil-specific *STAT3* knockout NStat3^−/−^ (*Ly6G*^*cre*^*STAT3*^*fl/fl*^) and WT (*Ly6G*^*wt*^*STAT3*^*fl/fl*^) mice throughout the study. For the experiments, female littermates between 8 and 12 weeks of age were used. The mice were housed and bred under specific pathogen-free conditions in cages of up to 5 mice per cage with a 12 h light/dark cycle at the animal facility of the University Hospital Essen. All the animal experiments were approved by the regulatory authorities LANUV (Das Landesamt für Natur, Umwelt und Verbraucherschutz Nordrhein-Westfalen, Germany). Our animal care and used protocols adhere to the regulations of German law according to das Deutsche Tierschutzgesetz (TierSchG) and follow the recommendations of the Federation of European Laboratory Animal Science Associations (FELASA).

The MOPC, MOPC^e-GFP^ and B16-F10 cells were injected subcutaneously (s.c. 1 × l 10^6^ in 100 µl of PBS) into the right flank of the mice.

The mice were injected intratumorally with 5 mg/kg CpG-Stat3ASO, CpG 1585 ODN (5 mg/kg) (InvivoGen, tlrl-1585) or PBS on day 5 posttumor injection. The CpG oligonucleotide conjugates were synthesized in the DNA/RNA synthesis core (COH) as previously described.^51^ For CD8^+^ T cell depletion, the mice were intraperitonally injected with an anti-CD8a antibody (250 μg, clone 2.43, Assay Genie) or an isotype control antibody (250 μg, Rat IgG2b, Assay Genie) on days −1, 0, 1, 8, and 15 after tumor injection.

### Flow cytometry of blood and tissue single-cell suspensions

Tissue samples (tumors and TDLNs) were digested in an enzyme mixture (as described above), and the cell pellet was resuspended in PBS containing mouse BD Fc block (1:500, BD Biosciences, clone: 2.4G2, RRID:AB_394656) or human BD Fc block (1:500 concentration, BD Biosciences, clone: Fc1.3216, RRID:AB_2728082) and incubated for 10 min at 20 °C. Whole blood (obtained by heart puncture with a heparin-coated needle and syringe) and single-cell suspensions were stained with eBioscience Fixable Viability Dye (Thermo Fisher Scientific, 65-0865-18) and specific antibodies. By staining for expression markers, appropriate isotype control antibodies were used in an additional reaction set. Staining was performed for 30 min at 4 °C. Stained human blood samples were also lysed with BD Pharm Lyse Lysing buffer (BD Biosciences), and mouse blood samples were subjected to ACK lysis buffer (150 mM NH4Cl, 10 mM KHCO3, and 0.1 mM Na2EDTA in ddH_2_O, pH 7.3). The data were recorded via the BD FACS Canto system and BD FACSymphony™ A5 Cell Analyzer (for high-parameter single-cell flow cytometry). The results were analyzed via FlowJo software. The following mouse antibodies were used in this study: anti-mouse EOMES (15321330, eBioscience™), anti-mouse CD62L (104433, BioLegend), anti-mouse CD11a/CD18 (LFA-1) (141013,BioLegend), anti-mouse CD11b (101219, BioLegend), anti-mouse CD11c (117339, BioLegend), anti-mouse CD14 (123313,BioLegend), anti-mouse CD152 (CTLA-4) (106323, BioLegend), anti-mouse CD182 (CXCR2) (750141, BD Biosciences), anti-mouse CD184 (CXCR4) (146511, BioLegend), anti-mouse CD197 (CCR7) (120121, BioLegend), anti-mouse CD206 (141705, BioLegend), anti-mouse CD223 (LAG-3) (741594, BD Biosciences), and anti-mouse CD274 (PD-L1) (746275. BD Biosciences), anti-mouse CD279 (PD-1) (752354, BD Biosciences), anti-mouse CD284/MD-2 complex (TLR4) (741015, BD Biosciences), anti-mouse CD289 (TLR9) (159107, BioLegend), anti-mouse CD3 (100215, BioLegend), anti-mouse CD4 (741218, BD Biosciences), anti-mouse CD45 (569151, BD Biosciences), anti-mouse CD54 (ICAM1) (740222, BD Biosciences), anti-mouse CD8 (563332, BD Biosciences), anti-mouse CD80 (741272, BD Biosciences), anti-mouse CD86 (751557, BD Biosciences), anti-mouse CXCL13 (17-7981-80, eBioscience™), anti-mouse F4/80 (123116, BioLegend), anti-mouse Granzyme B (372213, BioLegend), anti-mouse IFN-γ (505859, BioLegend), anti-mouse IL-10 (564081, BD Biosciences), anti-mouse IL-12 (505209, BioLegend), anti-mouse iNOS (53-5920-82, eBioscience), anti-mouse Ki-67 (564071, BD Biosciences), anti-mouse Ly-6C (128035, BioLegend), anti-mouse Ly-6G (741813, BD), anti-mouse MHC I (116616, BioLegend), anti-mouse MHC II (107614, BioLegend), anti-mouse Perforin (154405, Biolegend), anti-mouse STAT3 (MA5-23635, Invitrogen), anti-mouse STAT3 (phosho Tyr705) (651004, BioLegend), anti-mouse TIGIT (744213, BD Biosciences), anti-mouse TNF-α (506358, BioLegend), SYTOX™ Green Nucleic Acid Stain (S7020, ThermoFisher), Zombie UV™ Fixable Viability (423107, BioLegend). Human antibodies used in this study as followThe following human antibodies were used in this study: anti-human CD11b (101229, BioLegend), anti-human CD182 (CXCR2) (320704, BioLegend), anti-human CD284 (TLR4) (312811, BioLegend), anti-human CD284 (TLR4) (312811, BioLegend), anti-human CD3 (563797, BD Horizon), anti-human CD45 (568747, BD Biosciences), anti-human CD54 (ICAM1) (353116, BioLegend), anti-human CD62L (304826, BioLegend), anti-human CD66b (392915, BioLegend), anti-human CD8 (612755, BD Biosciences), anti-human CD80 (305206, BioLegend), anti-human CD86 (374206, BioLegend), anti-human CD95 (FasR) (752346, BD Biosciences), anti-human Granzyme B (563388, BD Biosciences), anti-human IFN-γ (502521, BioLegend), anti-human Ki-67 (151206, BioLegend), anti-human Perforin (308105, Biolegend), anti-human STAT1 (phosho Tyr701) (562070, BD Biosciences), anti-human STAT3 (MA5-23635, Invitrogen), anti-human STAT3 (phosho Tyr705) (651004, BioLegend)

### Fluorescence-activated cell sorting (FACS) of immune cells

Blood and tissue single-cell suspensions were incubated with PBS containing mouse FC block, anti-Ly6G, and anti-CD11b antibodies and viability dye for neutrophil isolation and with PBS containing anti-CD3 and anti-CD8 antibodies and viability dyes for CD8^+^ T cell isolation. The cells were sorted via a BD FACS Aria III Cell Sorter with a purity > 95%.

### Isolation of bone marrow neutrophils

Neutrophils were isolated from the bone marrow of naive mice. Bone marrow cells were collected by crushing the bone in a mortar under aseptic conditions. The cells were passed through 100 µm filters (Cell Trics, Partec, Sysmex), and erythrocytes were lysed in ACK buffer. Single-cell suspensions were stained with the antibodies listed below, Ly6G^+^ live neutrophils were sorted via a FACS Aria cell sorter (BD Biosciences), the purity of the cells was assessed, and all the neutrophils were CD11b^+^. After sorting, the cells were resuspended in DMEMc.

### Bulk RNA-seq

#### Sample preparation

Tumors were digested in an enzyme mixture (as described above), and the cell pellet was resuspended in PBS containing Mouse BD Fc Block (1:500, BD Biosciences, Clone: 2.4G2, RRID:AB_394656), anti-Ly6G, and anti-CD11b antibodies and viability dye for neutrophil isolation. The cells were sorted via a BD FACS Aria III Cell Sorter with a purity > 95%. Total RNA was isolated by using an RNeasy Micro Kit (50) (Qiagen, 74004).

### Sequencing and alignment

Low-input RNA analysis was performed after rRNA depletion. Total RNA was isolated and sequenced at 40 million paired-end reads. The raw FASTQ files were trimmed to 2 × 75 bp via seqtk and quantified via salmon (v1.6.0) against a GENCODE vM23 genome-decoyed transcriptome index with --validateMappings, --gcBias, and --posBias options enabled. Quantification was performed via a standardized pipeline (atpoint/rnaseq_preprocess), with sample metadata compiled into a samplesheet.csv. Transcript-level abundances were summarized at the gene level via tximport with the lengthScaledTPM method, enabling compatibility with downstream count-based differential expression analysis. The command lines and software versions are available in the pipeline_info directory. The resulting count table was imported into R (version 4.2.3) for downstream analysis.

### Loading and preparing count data

Gene-level counts were read via standard R functions (e.g., read.delim()). Genes with very low expression (total counts ≤ 10) were excluded from further analysis. Sample names were parsed to create a metadata table (coldata) that assigned each sample to its experimental condition.

### Data normalization and PCA

After the counts were imported, the counts were rounded to the nearest integer, and a DESeq2 (v1.42.1) dataset was constructed via the design formula “~ condition.” Prior to performing differential expression analyses, genes with low expression were filtered out. A variance-stabilizing transformation (VST) was applied (using vst(dds, blind = FALSE)) to reduce the dependence of variance on mean expression levels. Sample-to-sample distances were computed on the VST-transformed data and visualized as a heatmap (using pheatmap, v1.0.12) to evaluate overall similarity. Additionally, principal component analysis (PCA) was performed and visualized with ggplot2 (v3.5.1) to assess the clustering of samples by experimental conditions. Outlier detection was conducted by calculating the Mahalanobis distance on the first two principal components. Samples exceeding a chi-square threshold (with 2 degrees of freedom) were flagged as outliers and subsequently removed from the dataset. The PCA and sample distance heatmaps were recalculated after outlier removal to verify improved clustering. For heatmap visualization, logCPM values were scaled (rowwise z scores), and extreme values were clipped to the 5th and 95th percentiles. The pheatmap (v1.0.12) package was then used to plot the heatmap with rows (genes) clustered and columns (samples).

### Gene signature and pathway analysis

For custom gene set analysis, the raw count data were transformed into log counts per million (logCPM) values via edgeR (v4.0.16). Gene symbols were extracted from the count matrix, and an Excel file containing predefined gene sets was read via readxl (v1.4.5). For each gene set, the intersection between the set and the expressed genes was determined. A “signature score” was then calculated per sample as the mean logCPM of the genes in the set. The signature scores were compared between experimental conditions via two-sample t tests, and log2-fold changes were computed as the difference in the mean signature score (NSTAT3^−/−^–WT).

### Patient-derived tumor explants

HNC patient blood samples were drawn into 3.2% sodium citrate anticoagulant monovettes (Sarstedt) and mixed 1:1 with PBS (Gibco) before separation by density gradient centrifugation (Biocoll density of 1077 g/ml; Merck). The mononuclear cell fraction was discarded, and neutrophils were isolated by sedimentation over 1% polyvinyl alcohol, followed by hypotonic lysis (0.2% NaCl) of erythrocytes. Isolated neutrophils (1 mln/ml) were incubated with DMEMc ±LLL12 (an inhibitor of STAT3 phosphorylation) (2.5 nmol/mL) (573131, Merck) for 1 hour at 37 °C and 5% CO2. After incubation, the cells ( ~ 5 × 10^6^ per^1 mm3^ tumor) were washed with PBS and then resuspended in fresh DMEMc medium prior to coincubation with the tumor tissues obtained from the same HNC patients. These tumor tissues were first cut into two equal pieces, placed into 24-well cell culture plates and incubated together with the ±LLL12-stimulated PMNs overnight at 37 °C and 5% CO_2_. The culture media was removed, and the tumors were digested in the enzyme mixture as described above. The pelleted single cells were resuspended in DMEMc medium containing monensin and brefeldin A (1:1000, Biolegend) to block cytokine release from the cells and incubated in 24-well plates for 4 hours at 37 °C and 5% CO_2_. Furthermore, extra and intracellular immune-staining for flow cytometry was performed. The cells were washed with PBS, resuspended in PBS containing anti-CD45, anti-CD3, or anti-CD8 antibodies, viability dye and incubated at 4 °C for 30 min. The cells were subsequently washed with PBS, fixed and permeabilized via the FOXP3 Fix/Perm Buffer Set (BioLegend). to the manufacturer’s protocol. Washed cells were stained in 1× permeabilization buffer containing anti-IFN-γ, anti-GZMB, anti-Ki67 and anti-perforin antibodies, incubated at 4 °C for 30 min, washed and measured with a BD FACSCanto™ II Clinical Flow Cytometry System.

### Tumor killing assay with CD8^+^ T cells

Mononuclear cell fractions and neutrophils were isolated from patient blood via density gradient centrifugation as described above. Isolated neutrophils (1 million/ml) were incubated with DMEMc medium ±LLL12 (2.5 nmol/mL) for 1 h at 37 °C and 5% CO_2_. After incubation, the cells were washed with PBS and resuspended in fresh DMEMc medium before being cocultured with T cells. CD8^+^ T cells were sorted from the mononuclear fraction by using anti-CD3 and anti-CD8 antibodies and viability dyes. Neutrophils and CD8^+^ T cells were coincubated in 12-well cell culture plates at a 1:1 ratio overnight at 37 °C and 5% CO_2_. Next, these immune cells were seeded into new 12-well plates, and UT-SCC-50 cancer cells were added on top at a 1:1 (immune cell:tumor cell) ratio. The cells were incubated for 24 h at 37 °C and 5% CO_2_. To estimate the killing capacity of the pretreated CD8^+^ T cells on the tumor cells, the cell suspension (immune cells and dead tumor cells) was removed, and the wells were washed gently with PBS. To fix the viable tumor cells, 1 mL of ice-cold 96% methanol was added to each well and incubated for 10 min at −20 °C. The methanol was removed, and the plate was dried before 0.01% gentianine (PPF Hasko-Lek) was added to stain the tumor cells. After short staining, the cells were washed with dH_2_O and dried overnight. Images were taken with a professional camera using an Olympus CK2 bright-field microscope and analyzed with FIJI (ImageJ) software.

### Immunofluorescence

For histological examination of metastatic seeding in TDLNs, the mice were sacrificed on day 12 after tumor transplantation. TDLNs were dissected and snap frozen in liquid nitrogen in Tissue-Tek O.C.T. Compound (Sakura Finetek) containing 5% paraformaldehyde at −80 °C. 7-μm cryosections were mounted with Neo-Mount (Merck). For histopathological evaluation of HNC patient tumors, tissue microarrays (TMAs) were prepared from paraffin blocks at a thickness of 4 µm via Thermo Scientific Microm HM 340E. The samples were deparaffinized and rehydrated according to a standard protocol. Antigen retrieval was performed by using 1× citrate buffer (pH 6.0, 20× Concentrate, 00--5000, Invitrogen). Blocking was performed via the use of PBS containing 5% bovine serum albumin (BSA) (A7030, Merck) and 3% Triton X-100 (sc-29112, Santa Cruz Biotechnology). Anti-human CEACAM8 (10180794, Invitrogen) was used to detect neutrophils together with anti-human phospho-Stat3 (Tyr705) (4113, CST) and DAPI (4’,6-diamidino-2-phenylindole, dilactate) (422801, BioLegend). Microscopy was performed using a Zeiss AxioObserver. Z1 Inverted Microscope with ApoTome Optical Sectioning equipped with filters for: DAPI, FITC, Alexa Fluor 488, GFP, DsRed, Cy3 or Olympus BX51 upright epifluorescence microscope. The images were processed with ZEN Blue 2012 software and later analyzed with QuPath 0.5.1 software.

### Reactive oxygen species

After single-cell neutrophil suspensions from tumors were obtained, the cells were washed and resuspended in PBS containing FC-blocking, anti-Ly6G, and anti-CD11b antibodies and viability dye and then incubated at 4 °C for 30 min. Next, the cells were washed with PBS, resuspended in DMEMc containing ±PMA (5 ng/ml, Biomol, Cay10008014) and incubated for 1 hour at 37 °C and 5% CO_2_. ROS production by live CD11b^+^Ly6G^+^ neutrophils was estimated via flow cytometry with dihydrorhodamine 123 (Merck).

### Apoptosis assay

For the measurement of apoptosis, a FITC Annexin V Apoptosis Detection Kit with 7-AAD (BioLegend, 640922) was used according to the manufacturer’s protocol.

### Analysis of publicly available databases

The following available databases were analyzed: GSE83519 (GSM2205799, GSM2205800, GSM2205801, GSM2205802, GSM2205803, GSM2205804, GSM2205805, GSM2205806, GSM2205807, GSM2205808, GSM2205809, GSM2205810, GSM2205811, GSM2205812, GSM2205813, GSM2205814, GSM2205815, GSM2205816, GSM2205817, GSM2205818, GSM2205819, GSM2205820, GSM2205821, GSM2205822, GSM2205823, GSM2205824, GSM2205825, GSM2205826, GSM2205827, GSM2205828, GSM2205829, GSM2205830, GSM2205831, GSM2205832, GSM2205833, GSM2205834, GSM2205835, GSM2205836, GSM2205837, GSM2205838, GSM2205839, GSM2205840, GSM2205841, GSM2205842) (last date of assessment 10.08.2023), GSE79404 (GSM2094831, GSM2094832, GSM2094833, GSM2094834, GSM2094835, GSM2094836, GSM2094837, GSM2094838, GSM2094841, GSM2094842, GSM2094843, GSM2094844, GSM2094839, GSM2094840, GSM2094845, GSM2094846) (last date of assessment 10.08.2023), (PMID: 28417112), GSE122272 (GSM3462928, GSM3462935, GSM3462921, GSM3462944, GSM3462913, GSM3462932, GSM3462936, GSM3462941, GSM3462943, GSM3462922, GSM3462924, GSM3462925, GSM3462926, GSM3462933, GSM3462947, GSM3462919, GSM3462931, GSM3462945, GSM3462946, GSM3462914, GSM3462915, GSM3462916, GSM3462917, GSM3462920, GSM3462923, GSM3462927, GSM3462929, GSM3462930, GSM3462934, GSM3462937, GSM3462938, GSM3462939, GSM3462940, GSM3462942, GSM3462948, GSM3462949, GSM3462918) (last date of assessment 25.09.2023), (PMID: 31173964)

### Statistics

GraphPad Prism 10 (https://www.graphpad.com) was used to plot all the graphs and perform the statistical and quantitative assessments. Statistical analyses were performed via Kruskal‒Wallis ANOVA for multiple comparisons with the Bonferroni correction, the Mann‒Whitney *U* test for two independent samples, the Wilcoxon test for dependent samples and Student’s *t* test where indicated; correlations were analyzed with the Spearman *R* test. The Kaplan–Meier method was used to analyze tumor survival data, and the log-rank test was used for univariate analyses to compare survival curves for different groups. *P* < 0.05 was considered significant.

### Study approval

The animal experiments were approved by the regulatory authorities LANUV (Das Landesamt für Natur, Umwelt und Verbraucherschutz Nordrhein-Westfalen), Germany. Our animal care and used protocols adhere to the regulations of das Deutsche Tierschutzgesetz (TierSchG) and follow FELASA recommendations.

## Supplementary information

Neutrophil-specific targeting of STAT3 impairs tumor progression via the expansion of cytotoxic CD8+ T cells

Dataset 1

## Data Availability

The bulk RNA sequencing data have been deposited under the accession code GSE300511.
