# Supplementary material for: Neutrophil-specific targeting of STAT3 impairs tumor progression via the expansion of cytotoxic CD8+ T cells
Source: Signal Transduct Target Ther. 2025 Aug 30;10:279. doi: 10.1038/s41392-025-02363-z (PMC12398498; doi:10.1038/s41392-025-02363-z)
Supplement: Supplementary file 1 — Neutrophil-specific targeting of STAT3 impairs tumor progression via the expansion of cytotoxic CD8+ T cells [file 41392_2025_2363_MOESM1_ESM.pdf]

Supplementary Materials for

Neutrophil-specific targeting of STAT3 impairs tumor progression via the expansion of cytotoxic CD8$^+$ T cells

Irem Ozel, Guanyu Sha, Agnieszka Będzińska, Ekaterina Pylaeva, Yuliia Naumova, Ilona Thiel, Joanna Antczak, Anthony Squire, Matthias Gunzer, Gennadiy Zelinskyy, Cornelius Kürten, Stephan Lang, Carlos Silvestre-Roig, Marcin Kortylewski, Zvi Granot and Jadwiga Jablonska

Correspondence to: jadwiga.jablonska@uk-essen.de

This PDF file includes:

Materials and Methods
Figures. S1 to S5
Table S1

Materials and Methods

Flow cytometry of blood and tissue single cell suspension

Tissue samples (tumors and TDLNs) were digested in an enzyme solution (as described above) and the cell pellet was resuspended in PBS containing Mouse BD Fc Block (1:500, BD Biosciences, Clone: 2.4G2, RRID:AB_394656) or Human BD Fc Block (1:500 concentration, BD Biosciences, Clone: Fc1.3216, RRID:AB_2728082) and incubated for 10 min at 20 °C. Whole blood (obtained by heart punction with heparin coated needle and syringe) and tissue single-cell suspensions were stained with eBioscience Fixable Viability Dye (Thermo Fisher Scientific, 65-0865-18) and specific antibodies. By staining of expression markers appropriate isotype control antibodies were used in additional reaction set. Staining was performed for 30 min at 4 °C. Stained human blood samples were additionally lysed with BD Pharm Lyse Lysing buffer (BD Biosciences) and mouse blood samples with ACK lysis buffer (NH_4Cl 150 mM, $KHCO_3$ 10 mM, Na_2EDTA 0.1 mM, in ddH_2O, pH 7.3). Data were recorded using BD FACS Canto system and BD FACSymphony™ A5 Cell Analyzer (for high-parameter single cell flow cytometry)**.** Results were analyzed using FlowJo software. Mouse antibodies used in this study as follow: anti-mouse EOMES (15321330, eBioscience™), anti-mouse CD62L (104433, BioLegend), anti-mouse CD11a/CD18 (LFA-1) (141013, BioLegend), anti-mouse CD11b (101219, BioLegend), anti-mouse CD11c (117339, BioLegend), anti-mouse CD14 (123313,BioLegend), anti-mouse CD152 (CTLA-4) (106323, BioLegend), anti-mouse CD182 (CXCR2) (750141, BD Biosciences), anti-mouse CD184 (CXCR4) (146511, BioLegend), anti-mouse CD197 (CCR7) (120121, BioLegend) anti-mouse CD206 (141705, BioLegend), anti-mouse CD223 (LAG-3) (741594, BD Biosciences), anti-mouse CD274 (PD-L1) (746275. BD Biosciences), anti-mouse CD279 (PD-1) (752354, BD Biosciences), anti-mouse CD284/MD-2 Complex (TLR4) (741015, BD Biosciences), anti-mouse CD289 (TLR9) (159107, Biolegend), anti-mouse CD3 (100215, BioLegend), anti-mouse CD4 (741218, BD Biosciences), anti-mouse CD45 (569151, BD Biosciences), anti-mouse CD54 (ICAM1) (740222, BD Biosciences), anti-mouse CD8 (563332, BD Biosciences), anti-mouse CD80 (741272, BD Biosciences), anti-mouse CD86 (751557, BD Biosciences), anti-mouse CXCL13 (17-7981-80, eBioscience™), anti-mouse F4/80 (123116, BioLegend), anti-mouse Granzyme B (372213, BioLegend), anti-mouse IFN-γ (505859, BioLegend), anti-mouse IL-10 (564081, BD Biosciences), anti-mouse IL-12 (505209, Biolegend), anti-mouse iNOS (53-5920-82, eBioscience), anti-mouse Ki-67 (564071, BD Biosciences), anti-mouse Ly-6C (128035,

BioLegend), anti-mouse Ly-6G (741813, BD), anti-mouse MHC I (116616, BioLegend), anti-mouse MHC II (107614, BioLegend), anti-mouse Perforin (154405, Biolegend), anti-mouse STAT3 (MA5-23635, Invitrogen), anti-mouse STAT3 (phosho Tyr705) (651004, BioLegend), anti-mouse TIGIT (744213, BD Biosciences), anti-mouse TNF-α (506358, BioLegend), SYTOX™ Green Nucleic Acid Stain (S7020, ThermoFisher), Zombie UV™ Fixable Viability (423107, BioLegend). Human antibodies used in this study as follow: anti-human CD11b (101229, BioLegend), anti-human CD182 (CXCR2) (320704, BioLegend), anti-human CD284 (TLR4) (312811, BioLegend), anti-human CD284 (TLR4) (312811, BioLegend), anti-human CD3 (563797, BD Horizon), anti-human CD45 (568747, BD Biosciences), anti-human CD54 (ICAM1) (353116, BioLegend), anti-human CD62L (304826, BioLegend), anti-human CD66b (392915, BioLegend), anti-human CD8 (612755, BD Biosciences), anti-human CD80 (305206, BioLegend), anti-human CD86 (374206, BioLegend), anti-human CD95 (FasR) (752346, BD Biosciences), anti-human Granzyme B (563388, BD Biosciences), anti-human IFN-γ (502521, BioLegend), anti-human Ki-67 (151206, BioLegend), anti-human Perforin (308105, Biolegend), anti-human STAT1 (phosho Tyr701) (562070, BD Biosciences), anti-human STAT3 (MA5-23635, Invitrogen), anti-human STAT3 (phosho Tyr705) (651004, BioLegend)

Fluorescence-activated cell sorting (FACS) of immune cells

Blood and tissue single cell suspensions were incubated with PBS containing Mouse FC-block, anti-Ly6G, anti-CD11b antibodies and viability dye for neutrophil isolation and PBS containing anti-CD3, anti-CD8 antibodies and viability dyes for $CD8^+$ T cell isolation. Cells were sorted using BD FACS Aria III Cell Sorter with the purity >95%.

Isolation of bone marrow neutrophils

Neutrophils were isolated from bone marrow of naive mice. Bone marrow cells were collected via crushing the bone in a mortar under aseptic conditions. Cells were meshed through 100µm filters (Cell Trics, Partec, Sysmex) and erythrocytes lysed in ACK buffer. Single-cell suspensions were stained with antibodies listed below, $Ly6G^+$ alive neutrophils were sorted using a FACS Aria cell sorter (BD Biosciences), and the purity of cells was assessed, all neutrophils are $CD11b^+$. After sorting cells were resuspended in DMEMc.

Migration assay

Migration of isolated neutrophils was evaluated using a two-chamber transwell system (3 µm pore size) cell culture inserts (Corning). For detection of spontaneous migration DMEMc medium was added to the lower chamber. Isolated bone marrow neutrophils $1x10^6$ cells in DMEMc, were added to the upper chamber and placed into medium for 3 h at 37 °C and 5% CO_2. Cells transmigrated to the lower chamber were counted using CASY system (Roche Innovatis).

Apoptosis assay

For the measurement of apoptosis, FITC Annexin V Apoptosis Detection Kit with 7-AAD (BioLegend, 640922) was used according to manufacturer's protocol.

Phagocytosis assay

For the measurement of the phagocytic process of Neutrophils, FITC-labelled beads from the Cayman's Phagocytosis Assay Kit (IgG FITC) (Cayman Chemical, 500290) were used according to manufacturer's protocol.

Cy3-CpG-ASO uptake

Neutrophils were sorted from MOPC tumors on day 10 and incubated with 5µM and 20µM Cy3-CpGASO for 30 min. Flow cytometry analysis was performed, results were analyzed using FlowJo software.

NETs release

Isolated neutrophils 15.000/well were incubated with ± PMA (5 ng/ml, Biomol, Cay10008014) in glass-bottom 96-well plate (MatTek Corporation) pre-coated with poly-D-lysine 1 mg/ml (Sigma-Aldrich) for 4 hours at 37 °C, 5% CO_2. Samples were fixed with paraformaldehyde (Thermo Fisher Scientific) to the final concentration of 4%, permeabilized with 0.2% Triton X-100 (Sigma-Aldrich) containing buffer. Anti-histone 1 antibody (Merck) is used to detect histone proteins on NETs. Donkey-anti-mouse-AF564 (Thermo Fisher Scientific) were used as secondary antibodies. Stainings were mounted with ProLong Gold Antifade Mountant with DAPI (Invitrogen). Percent of NET-producing cells, NETs length and area were estimated by microscopy followed by analysis with FIJI (ImageJ) software.

For the flow cytometry analysis of NET formation, blood was collected from mice after sacrificing via heart puncture in heparinized tubes. White blood cells were collected from blood after threefold lysis of red blood cells with ACK buffer containing NH_4Cl 150 mM, $KHCO_3$ 10 mM, Na_2EDTA

0.1 mM. Cells were washed with PBS and then incubated with Sytox Green, viability dyes, anti-Ly6G and anti-CD11b antibodies for 30 min at 20 °C. Stained cells were washed with PBS and measured by flow cytometry.

Western blot

Bone marrow neutrophils were lysed with RIPA buffer (50 mM Tris, pH 8 .0, 150 mM NaCl, 1% Triton® X-100, 0 .5% Sodium deoxycholate, 0 .1% SDS and freshly added protease inhibitors (Protease Inhibitor Cocktail Set I (Calbiochem, 539131), Protease Inhibitor Cocktail Set III (Calbiochem, 539134), PhosSTOP™ (Roche, 4906845001)) Cell lysates were separated by 10% SDS-PAGE (sodium dodecylsulfate polyacrylamide gel electrophoresis). Proteins were transferred on 0.45 µM nitrocellulose membranes and blocked with 5% skim milk in PBST for 1h at room temperature. After blocking the membranes were probed over night at 4 °C with the primary mouse anti-mouse Stat3 (CST, 9139) and rabbit anti-mouse Gapdh (as housekeeping protein, CST, 2118) antibodies. Membranes were incubated with appropriate horseradish peroxidase (HRP) conjugated secondary antibodies (115-035-003, Dianova), allowing signal visualization with Pierce ECL Western Blotting Substrate (Thermo Fischer Scientific, 32106). Signals were detected by Amersham Imager 600 and later quantified using FIJI (ImageJ) software.

Aortic ring assay

Thoracic aortae from C57Bl/6J WT mice were dissected, fat and connective tissue were removed and 0.5 mm aorta rings were placed in 96-well plates coated with type I collagen (Enzo life sciences). Once embedded, rings were covered with the endothelial cell growth medium (Promocell). Subsequently, 2×10^4 bone marrow neutrophils isolated from WT and $NStat3^{-/-}$ mice were added and incubated at 37 °C, 5% CO_2. The aortic rings with progressive sprouting were imaged every day for 14 days using AMG EVOS digital phase contrast inverted microscope and analyzed by FIJI (ImageJ) software.

Analysis of public available databases

The following available databases were analysed: GSE114445 (GSM3141862, GSM3141863, GSM3141864, GSM3141865, GSM3141866, GSM3141867, GSM3141868, GSM3141869, GSM3141870, GSM3141871, GSM3141872, GSM3141873, GSM3141874, GSM3141875,

GSM3141876, GSM3141877, GSM3141823, GSM3141824, GSM3141825, GSM3141826, GSM3141827, GSM3141828) (last date of assessment 25.04.2024) (PMID: 30326165).

Statistics

GraphPad Prism 10 (https://www.graphpad.com) was used to plot all graphs and to perform statistical and quantitative assessments. Statistical analyses were performed using Kruskal-Wallis ANOVA for multiple comparisons with the Bonferroni correction, and Mann-Whitney U-test for two independent samples, and Whilcoxon test for dependent samples and Student's T Test was used where indicated; correlations were analyzed with Spearman R test. The Kaplan–Meier method was used to analyze tumor survival data, and the log-rank test was used for univariate analyses to compare survival curves for different groups. $P<0.05$ was considered significant.

Study approval

The animal experiments have been approved by the regulatory authorities LANUV (Das Landesamt für Natur, Umwelt und Verbraucherschutz Nordrhein-Westfalen), Germany. Our animal care and used protocols adhere to the regulations of das Deutsche Tierschutzgesetz (TierSchG) and follow FELASA recommendations.

Figure. S1.

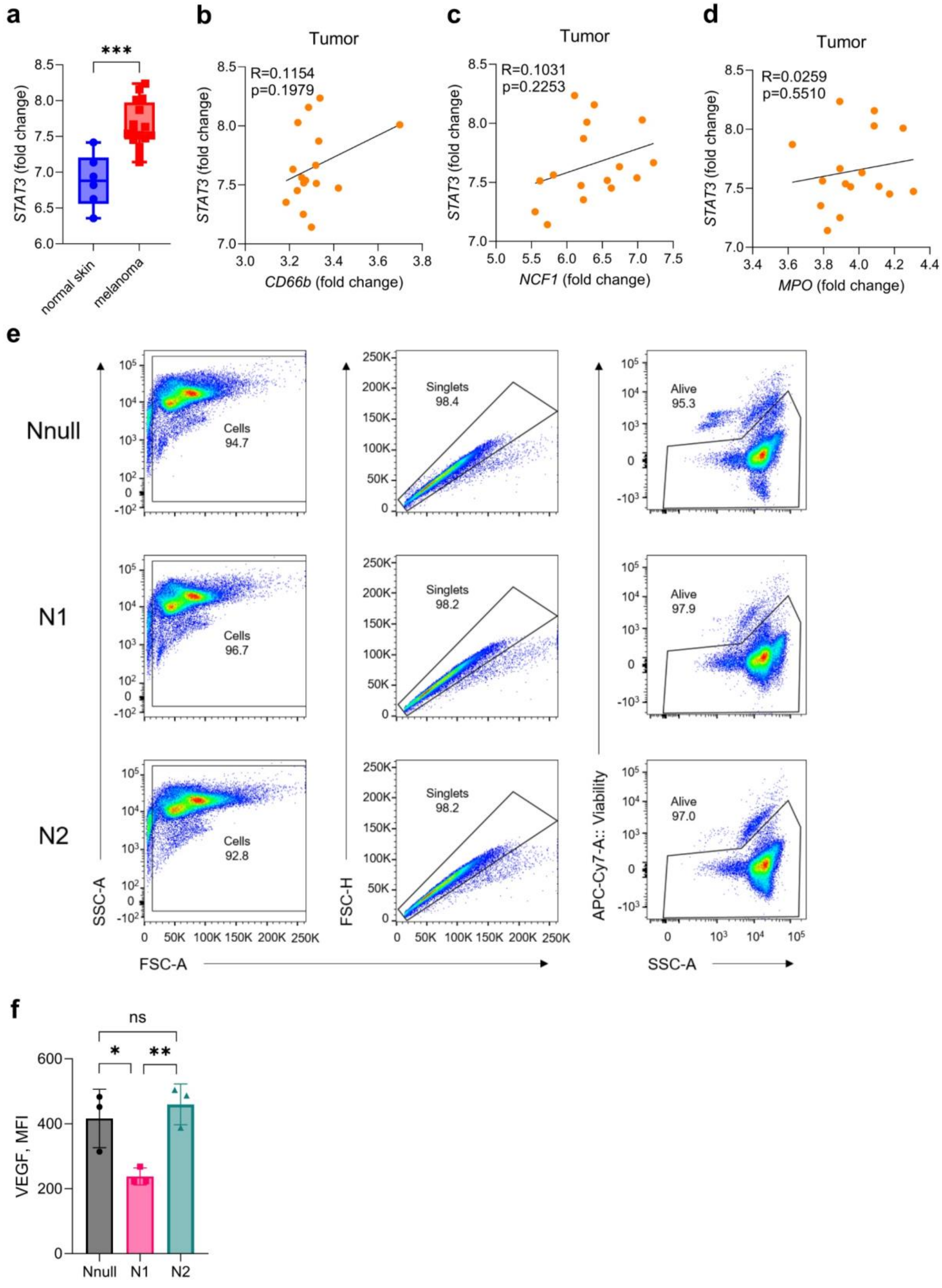

a
***
STAT3 (fold change)
normal skin
melanoma
b
Tumor
R=0.1154
p=0.1979
CD66b (fold change)
c
Tumor
R=0.1031
p=0.2253
NCF1 (fold change)
d
Tumor
R=0.0259
p=0.5510
MPO (fold change)
e
Nnull
N1
N2
Cells 94.7
Cells 96.7
Cells 92.8
Singlets 98.4
Singlets 98.2
Singlets 98.2
Alive 95.3
Alive 97.9
Alive 97.0
SSC-A
FSC-A
FSC-H
APC-Cy7-A:: Viability
SSC-A
f
ns
*
**
VEGF, MFI
Nnull
N1
N2

Supplementary Fig. 1. High STAT3 expression positively correlates with TANs in melanoma. (a) Analysis of transcriptomics data from GEO dataset GSE114445, showing STAT3 mRNA expression in fold changes comparing normal skin (*blue*) and melanoma (*red*). **(b)** Correlation analysis of transcription levels (in fold changes) of *STAT3* and *CD66b* in tumor tissues. Data analyzed from GEO dataset GSE114445. **(c)** Correlation analysis of transcription levels (in fold changes) of *STAT3* and neutrophil cytosolic factor 1 (*NCF1*) in tumor tissues. Data analyzed from GEO dataset GSE114445. **(d)** Correlation analysis of transcription levels (in fold changes) of *STAT3* and myeloperoxidase (*MPO*) in tumor tissues. Data analyzed from GEO dataset GSE114445. **(e)** Gating strategy of healthy blood neutrophils *in vitro* polarized into pro- (N2) and antitumoral (N1) states and left non-polarized (Nnull). **(f)** Bar graphs showing the MFI of VEGF in Nnull (*black*), N2 (*blue*) and N1 (*pink*) neutrophils. Statistical analyses were performed using Mann-Whitney-Test. For the correlation analysis, statistical significance and coefficient of determination were calculated using a Pearson correlation test. *P < 0.05, **P < 0.01, ***P < 0.001, ****P < 0.0001.

Figure. S2.

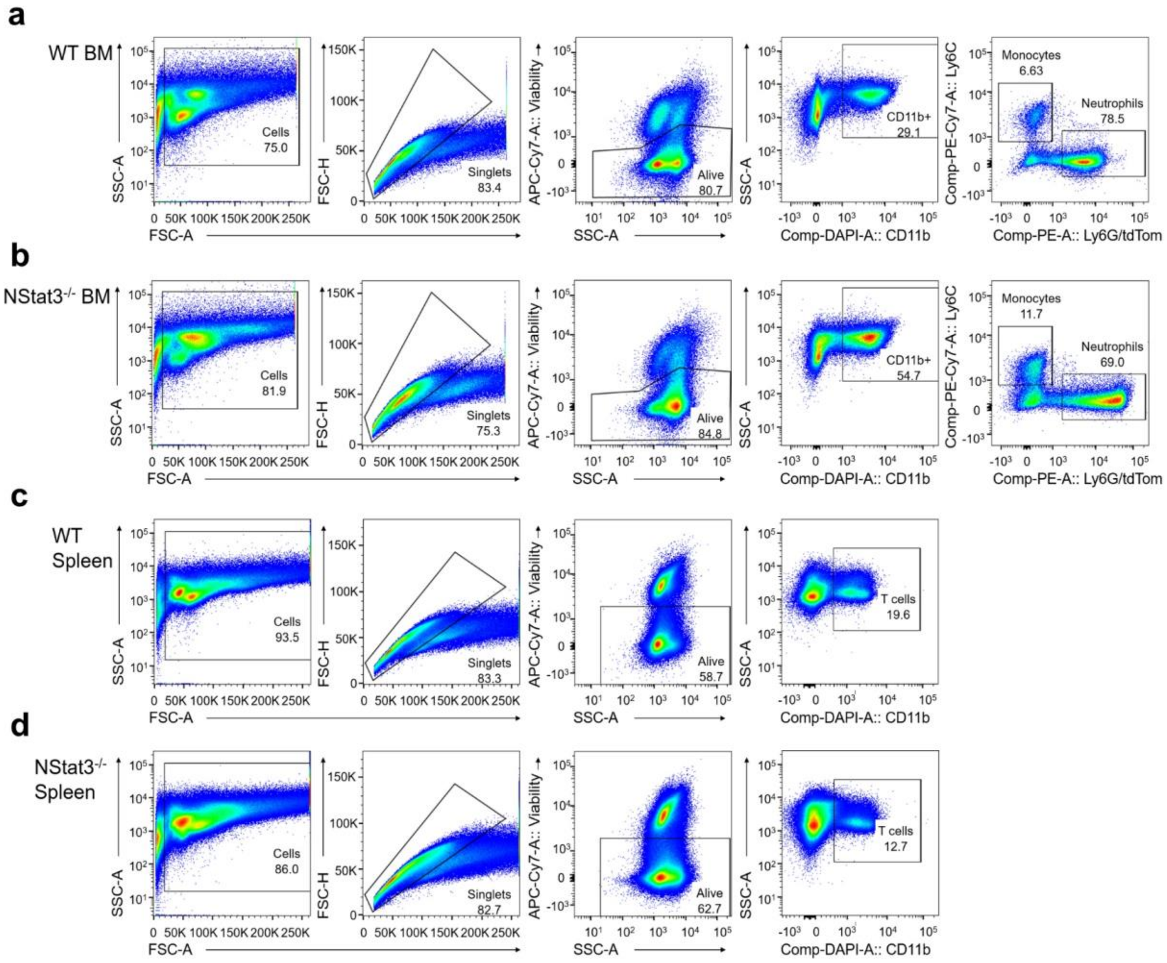
a
WT BM
Cells
75.0
Singlets
83.4
Alive
80.7
CD11b+
29.1
Monocytes
6.63
Neutrophils
78.5
SSC-A
FSC-A
FSC-H
APC-Cy7-A:: Viability
Comp-DAPI-A:: CD11b
Comp-PE-Cy7-A:: Ly6C
Comp-PE-A:: Ly6G/tdTom
b
NStat3-/- BM
Cells
81.9
Singlets
75.3
Alive
84.8
CD11b+
54.7
Monocytes
11.7
Neutrophils
69.0
c
WT
Spleen
Cells
93.5
Singlets
83.3
Alive
58.7
T cells
19.6
d
NStat3-/-
Spleen
Cells
86.0
Singlets
82.7
Alive
62.7
T cells
12.7

Supplementary Fig. 2. Gating strategy used for flow cytometry-based cell isolation of neutrophils, monocytes and T cells. Bone marrow monocytes & neutrophils and spleen T cells isolated from naïve WT and NStat3$^{-/-}$ mice. **(a)** Dot plots showing the gating strategy for bone marrow monocytes and neutrophils from naïve WT mouse. **(b)** Dot plots showing the gating strategy for bone marrow monocytes and neutrophils from naïve NStat3$^{-/-}$ mouse. **(c)** Dot plots showing the gating strategy for spleen T cells from naïve WT mouse. **(d)** Dot plots showing the gating strategy for spleen T cells from naïve NStat3$^{-/-}$ mouse.

Figure. S3.

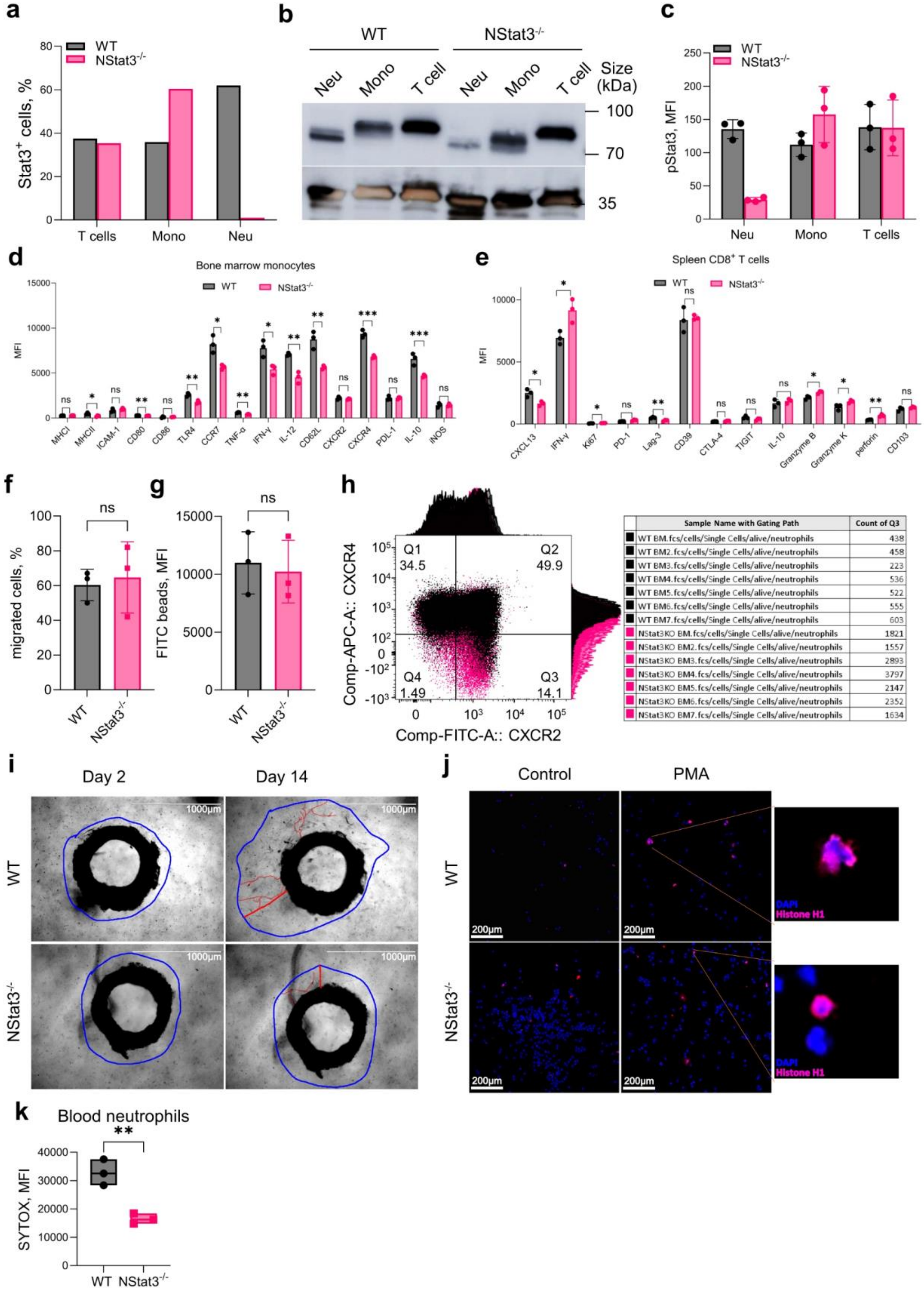

	Sample Name with Gating Path	Count of Q3
■	WT BM.fcs/cells/Single Cells/alive/neutrophils	438
■	WT BM2.fcs/cells/Single Cells/alive/neutrophils	458
■	WT BM3.fcs/cells/Single Cells/alive/neutrophils	223
■	WT BM4.fcs/cells/Single Cells/alive/neutrophils	536
■	WT BM5.fcs/cells/Single Cells/alive/neutrophils	522
■	WT BM6.fcs/cells/Single Cells/alive/neutrophils	555
■	WT BM7.fcs/cells/Single Cells/alive/neutrophils	603
■	NStat3KO BM.fcs/cells/Single Cells/alive/neutrophils	1821
■	NStat3KO BM2.fcs/cells/Single Cells/alive/neutrophils	1557
■	NStat3KO BM3.fcs/cells/Single Cells/alive/neutrophils	2893
■	NStat3KO BM4.fcs/cells/Single Cells/alive/neutrophils	3797
■	NStat3KO BM5.fcs/cells/Single Cells/alive/neutrophils	2147
■	NStat3KO BM6.fcs/cells/Single Cells/alive/neutrophils	2352
■	NStat3KO BM7.fcs/cells/Single Cells/alive/neutrophils	1634

Supplementary Fig. 3. Targeting STAT3-signaling modifies the tumor-related functions of neutrophils in mice. BM neutrophils isolated from WT and NStat3$^{-/-}$ mice. **(a)** Bar graph showing the neutrophil-specific Stat3 knockdown of NStat3$^{-/-}$ mice by flow cytometer. **(b)** Immunoblotting images showing the neutrophil-specific Stat3 knockdown of NStat3$^{-/-}$ mice by western blot. **(c)** Bar graph showing the neutrophil-specific pStat3 knockdown of NStat3$^{-/-}$ mice by flow cytometer. **(d)** Bar graph showing the MFI of indicated markers on naïve bone marrow monocytes from WT (*black*) and NStat3$^{-/-}$ (*pink*) mice. Markers measured by flow cytometer. **(e)** Bar graph showing the MFI of indicated markers on naïve spleen CD8^{+} T cells from WT (*black*) and NStat3$^{-/-}$ (*pink*) mice. Markers measured by flow cytometer. **(f)** Migration assay showing the percentage of WT (*black*) and NStat3$^{-/-}$ (*pink*) neutrophils migrated through Transwell membrane inserts. **(g)** Phagocytosis assay showing FITC signals from phagocytosed beads as MFI in WT (*black*) and NStat3$^{-/-}$ (*pink*) neutrophils measured by flow cytometry. **(h)** Dot plot with adjacent histograms of flow cytometry analysis showing the CXCR2 and CXCR4 expression on neutrophils from WT (*black*) and NStat3$^{-/-}$ (*pink*) mice (*left panel*). Statistical table of flow cytometry analysis on the left showing the count of neutrophils with CXCR2^{+}CXCR4^{-} phenotype in each measured sample (*right panel*). **(i)** Microscope images of aortic ring assay showing the neutrophil mediated new blood vessel formation and sprouting on day 2 and day 14. **(j)** Immunofluorescence of neutrophils from WT and NStat3$^{-/-}$ mice showing the presence of NETs with or without PMA stimulation. **(k)** Bar graph showing the MFI of SYTOX on blood neutrophils isolated from tumor-bearing WT (*black*) and NStat3$^{-/-}$ (*pink*) mice measured by flow cytometry on day 21 post-tumor injection. Statistical analyses were performed using Student's T Test. *P < 0.05, **P < 0.01, ***P < 0.001, ****P < 0.0001.

Figure. S4.

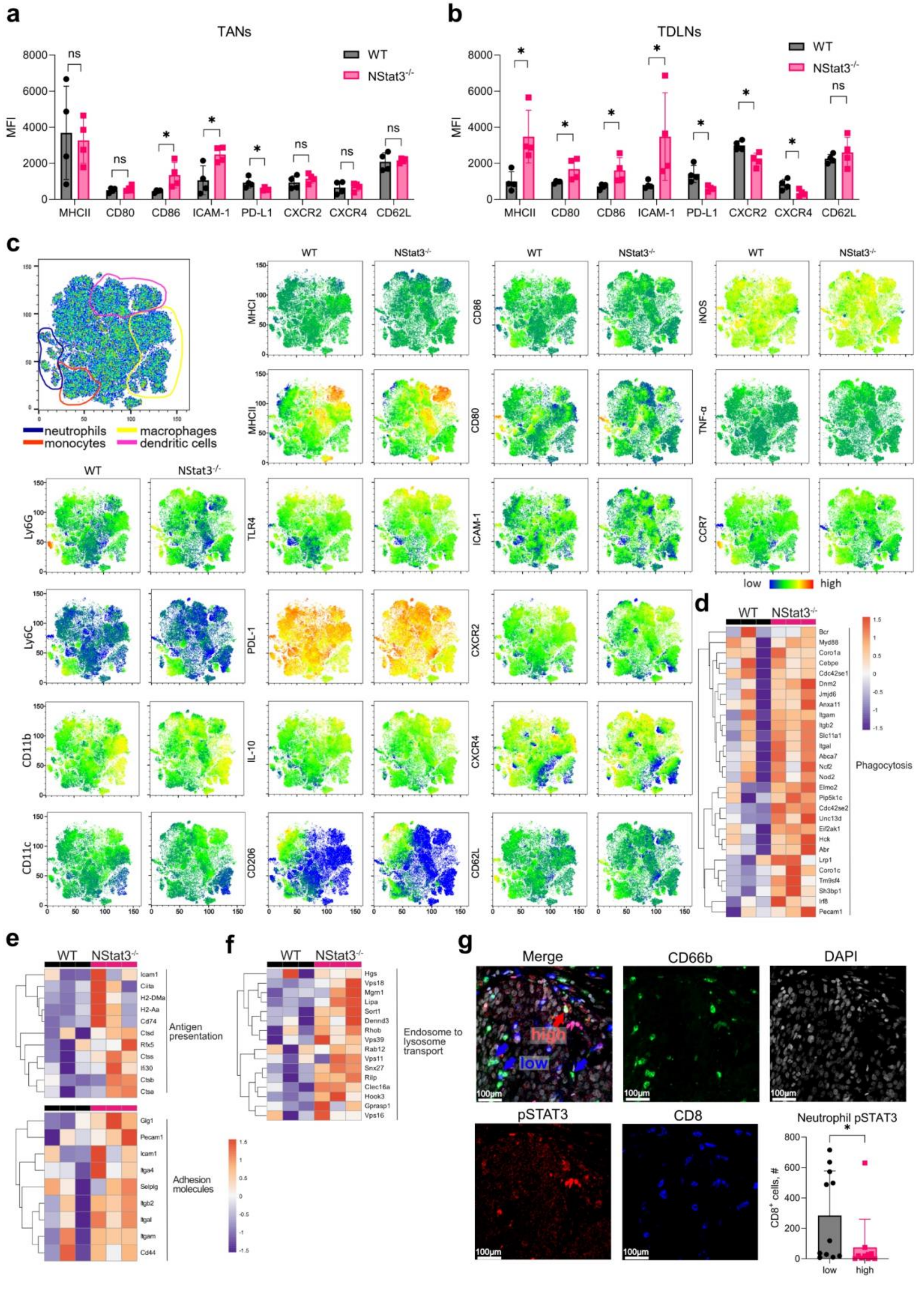
a
TANs
WT
NStat3-/-
MFI
ns
*
MHCII
CD80
CD86
ICAM-1
PD-L1
CXCR2
CXCR4
CD62L
b
TDLNs
c
neutrophils
monocytes
macrophages
dendritic cells
MHCI
MHCII
CD86
CD80
iNOS
TNF-α
Ly6G
Ly6C
CD11b
CD11c
TLR4
PD-L1
IL-10
CD206
ICAM-1
CXCR2
CXCR4
CD62L
CCR7
low
high
d
Phagocytosis
e
Antigen presentation
Adhesion molecules
f
Endosome to lysosome transport
g
Merge
CD66b
DAPI
pSTAT3
CD8
Neutrophil pSTAT3
CD8+ cells, #
100μm

Supplementary Fig. 4. Neutrophil-specific STAT3 deficiency alters myeloid cell landscape in tumors. WT and $NStat3^{-/-}$ mice were injected with B16-F10 as described in Fig 2a. Bar graph showing the expression of indicated markers on **(a)** TANs and **(b)** neutrophils in TDLNs from WT (*black*) and $NStat3^{-/-}$ (*pink*) mice. **(c)** WT and $NStat3^{-/-}$ mice were injected with MOPC as described in Fig 2a. Tumors analyzed by high-parameter single cell flow cytometry. Myeloid cells gated by their surface expression of CD11b (*macrophages*), CD11c (*dendritic cells*), Ly6G (*neutrophils*) and Ly6C (*monocytes*). tSNE plots showing the expression of indicated markers in tumor tissues from WT (*left panel*) and $NStat3^{-/-}$ (*right panel*) mice. **(d-f)** Heatmaps showing altered expression of genes related to **(d)** phagocytosis, **(e)** antigen presentation and adhesion molecules **(f)** endosome-to-lysosome transport from bulk RNA-seq of the TANs from WT (*black*) and $NStat3^{-/-}$ (*pink*) mice. The color scale indicates the log2 fold change in transcripts per million (TPM) for each gene, calculated as the ratio to the average TPM of control samples (n = 3 biological replicates). **(g)** Representative images of immunofluorescence of tumor tissues from HNC patients showing high (*red arrow*) and low (*blue arrow*) pSTAT3 expression in TANs, together with the presence of $CD8^+$ T cells, scale bars, 100 μm. Bar graph (*bottom right*) showing the number of $CD8^+$ cells in the same tumor tissue with high and low pSTAT3 expressing neutrophils. Statistical analyses were performed using Student's T Test. *P < 0.05, **P < 0.01, ***P < 0.001, ****P < 0.0001.

Figure. S5.

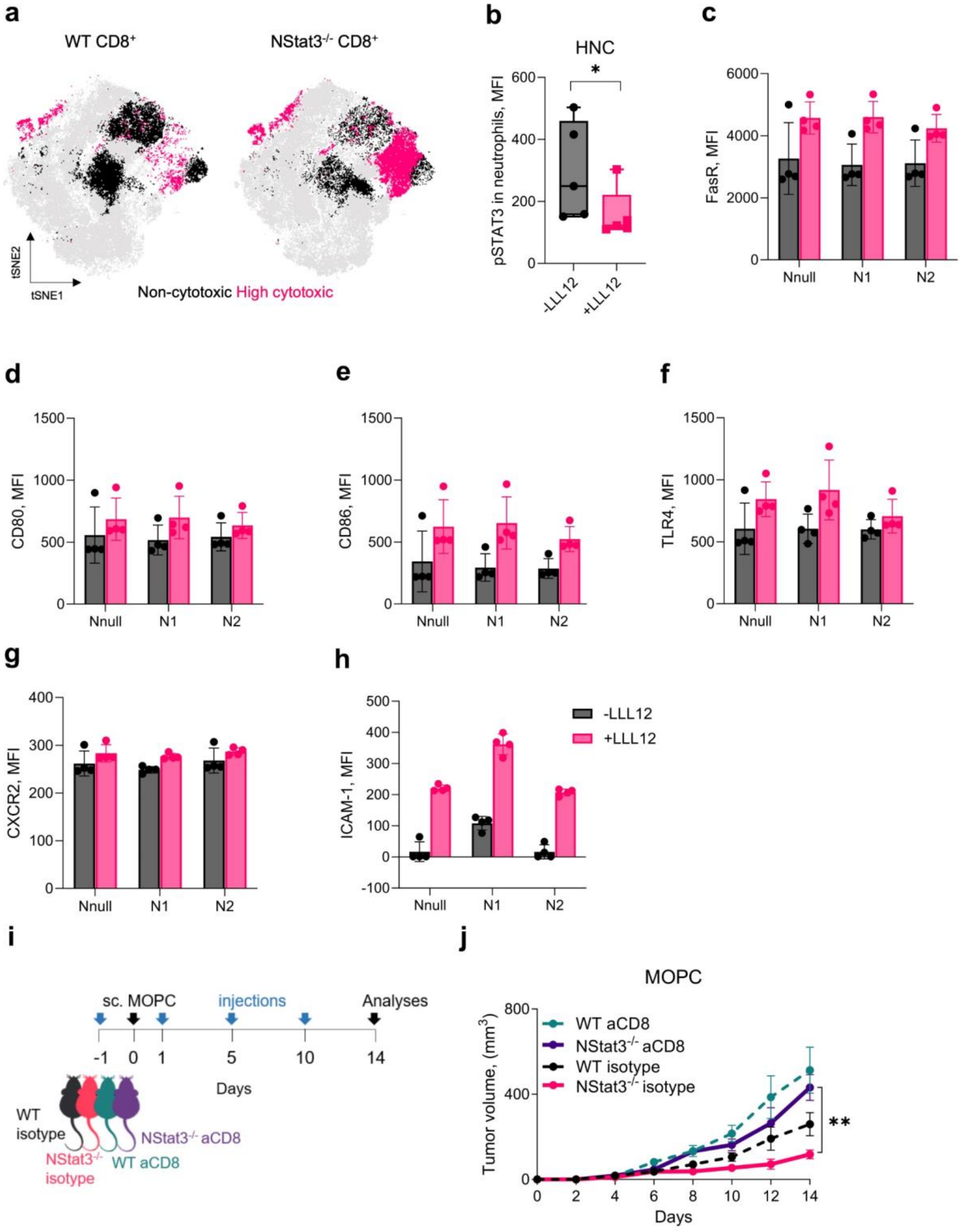
a
WT CD8⁺
NStat3-/- CD8⁺
tSNE2
tSNE1
Non-cytotoxic High cytotoxic
b
HNC
pSTAT3 in neutrophils, MFI
-LLL12
+LLL12
c
FasR, MFI
Nnull
N1
N2
d
CD80, MFI
e
CD86, MFI
f
TLR4, MFI
g
CXCR2, MFI
h
ICAM-1, MFI
-LLL12
+LLL12
i
sc. MOPC
injections
Analyses
Days
WT isotype
NStat3-/- isotype
WT aCD8
NStat3-/- aCD8
j
MOPC
Tumor volume, (mm3)
WT aCD8
NStat3-/- aCD8
WT isotype
NStat3-/- isotype
Days

Supplementary Fig. 5. Stat3-deficient neutrophils support cytotoxic T cell responses in TDLNs. WT and NStat3$^{-/-}$ mice were injected with MOPC as described in Fig 2a. **(a)** tSNE plots of CD8^{+} T cells of TDLNs isolated from WT (*left panel*) and NStat3$^{-/-}$ (*right panel*) mice. Colors indicate the IFN-γ^{lo}Ki67^{lo}GZMB^{lo}perforin^{lo} (*black*) and IFNγ^{hi}Ki67^{hi}GZMB^{hi}perforin^{hi} (*pink*) populations obtained by high-parameter single cell flow cytometry. **(b)** Blood neutrophils from HNC patients were isolated and treated with (*pink*) or without (*black*) LLL12. **(c-h)** Blood neutrophils from healthy donors were isolated and *in vitro* polarized into N1 and N2. Bar graph of **(c)** FasR, **(d)** CD80, **(e)** CD86, **(f)** TLR4, **(g)** CXCR2 and **(h)** ICAM-1 expression of healthy blood neutrophils treated with (*pink*) or without (*black*) LLL12. **(i)-(j)** Antitumor effect of Stat3-deficient neutrophils is CD8 T cell dependent. **(i)** Schematic representation of the experimental system, tumor cells were injected s.c., anti-CD8 antibody were injected intraperitonally into WT (*blue*) and NStat3$^{-/-}$ mice (*purple*), or isotype control into WT (*black*) and NStat3$^{-/-}$ (*pink*) mice. **(j)** Tumor growth curves showing the average tumor volume in intraperitonal anti-CD8 antibody injection in WT (*blue*) and NStat3$^{-/-}$ mice (*purple*) and isotype control antibody injection in WT (*black*) and NStat3$^{-/-}$ (*pink*) mice after MOPC injection (n = 3 mice per group). Statistical analyses were performed using Student's T Test. *P < 0.05, **P < 0.01, ***P < 0.001, ****P < 0.0001.

Figure. S6.

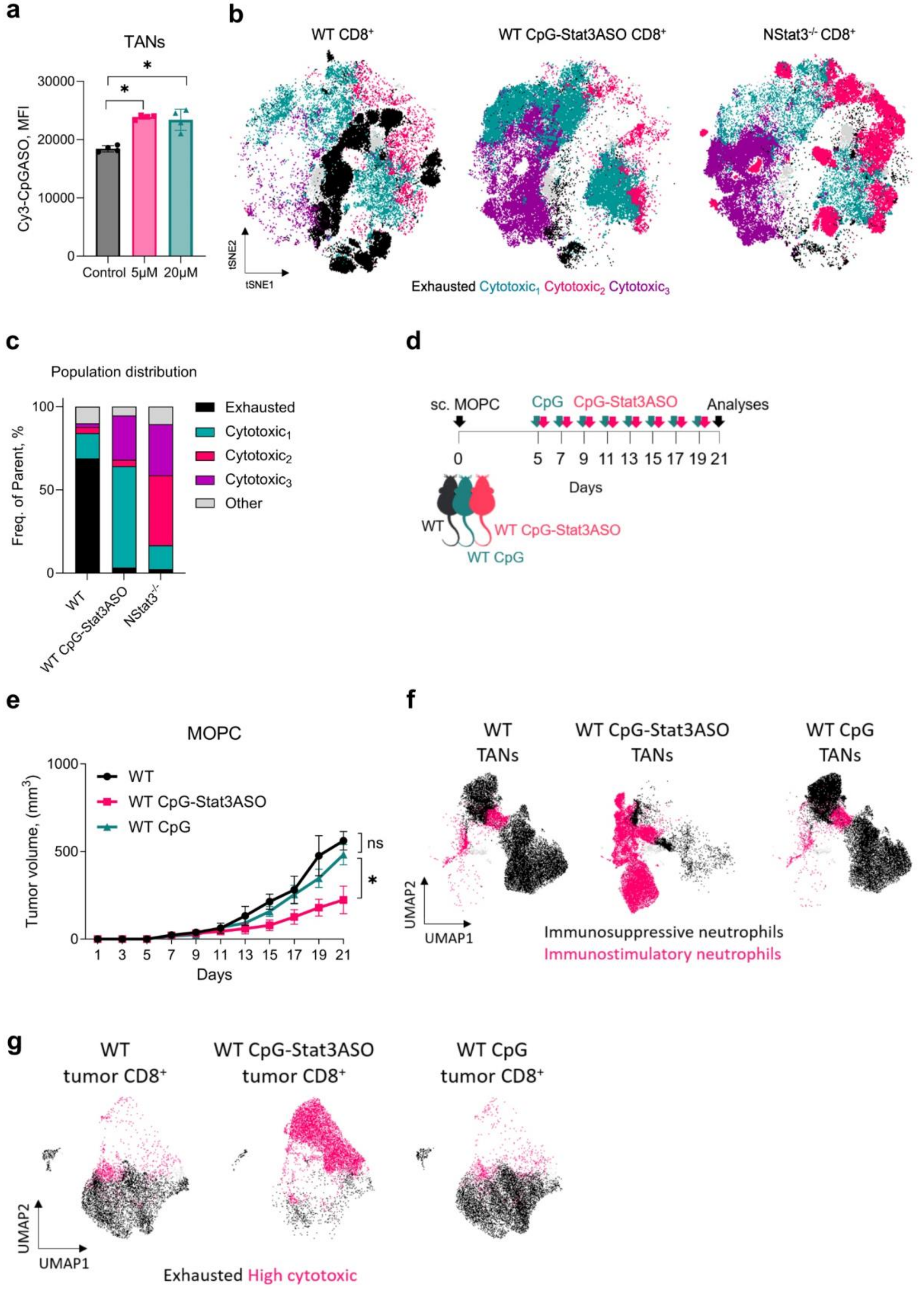
a
TANs
Cy3-CpGASO, MFI
30000
20000
10000
0
Control 5μM 20μM
b
WT CD8+
WT CpG-Stat3ASO CD8+
NStat3-/- CD8+
tSNE2
tSNE1
Exhausted Cytotoxic1 Cytotoxic2 Cytotoxic3
c
Population distribution
Freq. of Parent, %
100
50
0
Exhausted
Cytotoxic1
Cytotoxic2
Cytotoxic3
Other
WT
WT CpG-Stat3ASO
NStat3-/-
d
sc. MOPC
CpG CpG-Stat3ASO
Analyses
0 5 7 9 11 13 15 17 19 21
Days
WT
WT CpG
WT CpG-Stat3ASO
e
MOPC
Tumor volume, (mm3)
1000
500
0
WT
WT CpG-Stat3ASO
WT CpG
ns
*
1 3 5 7 9 11 13 15 17 19 21
Days
f
WT TANs
WT CpG-Stat3ASO TANs
WT CpG TANs
UMAP2
UMAP1
Immunosuppressive neutrophils
Immunostimulatory neutrophils
g
WT tumor CD8+
WT CpG-Stat3ASO tumor CD8+
WT CpG tumor CD8+
UMAP2
UMAP1
Exhausted High cytotoxic

Supplementary Fig. 6. Targeting STAT3 in TANs with CpG-Stat3ASO leads to the enrichment of effectory CD8^{+} T cells in TDLNs. WT and NStat3$^{-/-}$ mice were injected with MOPC as described in Fig 2a. **(a)** Bar graphs showing the Cy3-CpGASO uptake in mouse neutrophils after incubation with 5 µM (*pink*) and 20 µM (*blue*) for 30 min. **(b)** tSNE plots and **(c)** Stacked bar graphs of CD8^{+} T cells of TDLNs isolated from WT (*left panel*), CpG-Stat3ASO (*middle panel*) and NStat3$^{-/-}$ (*right panel*) mice. Colors indicate the IFN-γ^{lo}Ki67^{lo}PD-1^{hi}LAG-3^{hi}GZMB^{lo}GZMK^{lo}CTLA^{hi}TIGIT^{hi} (*black*), IFN-γ^{int}Ki67^{int}PD-1^{lo}LAG-3^{lo}GZMB^{hi}GZMK^{int}perforin^{int} (*blue*), IFN-γ^{hi}Ki67^{hi}CXCL13^{hi}PD-1^{int}GZMB^{hi}GZMK^{hi}perforin^{hi} (*pink*) and IFN-γ^{lo}Ki67^{int}PD-1^{lo}GZMB^{hi}GZMK^{hi}perforin^{hi}EOMES^{hi} (*purple*) populations obtained by high-parameter single cell flow cytometry. **(d)** Schematic representation of the experimental tumor model used for intratumoral CpG (*blue*) and CpG-Stat3ASO (*pink*) injection experiments. **(e)** Tumor growth curves showing the average tumor volume in WT (*black*), intratumoral CpG (*blue*) and CpG-Stat3ASO injected (*pink*) mice after MOPC injection (n = 3 mice per group). **(f)** UMAPs of TANs isolated from WT, WT CpG and WT CpG-Stat3ASO injected mice. Colors indicate the CXCR4^{hi}PDL-1^{hi}CD62L^{hi}IL10^{hi} (*black*) and MHCI^{hi}MHCII^{hi}ICAM1^{hi}CCR7^{hi}CD86^{hi} (*pink*) populations obtained by high-parameter single cell flow cytometry. **(g)** UMAP plots of tumor infiltrating CD8^{+} T cells isolated from WT (*left panel*) and NStat3$^{-/-}$ (*right panel*) mice. Colors indicate the PD-1^{hi}LAG-3^{hi}CD39^{hi}CTLA-4^{hi}TIGIT^{hi} (*black*) and IFN-γ^{hi}GZMB^{hi}GZMK^{hi} (*pink*) populations obtained by high-parameter single cell flow cytometry.

Table S1.

Clinicopathologic characteristics of volunteers who participated in the study (flow cytometry)

Patient #	Gender	Age at first diagnosis date	Tumor localisation	Tumor Stage	Nodal Status	Distant metastasis	HPV status	Smoking	Alcohol
2843	m	72	Larynx	T1	N0	M0	n/a	never	2-4x/month
2845	m	51	Oropharynx	T3	N0	M0	n/a	25py	no
2846	m	69	Larynx	T3	N2a	n/a	n/a	in the past	no
2847	m	68	Oropharynx	T2	N0	M0	p16-	50py	> 4x/week
2849	f	68	Larynx	T4	N3b	M0	n/a	40py	> 4x/week
2850	m	72	Larynx	T3	N0	M0	n/a	75py	chronic abusus
2868	m	71	Larynx	T2	N0	M1	p16-	60py	n/a
2873	m	46	Larynx	T4a	N2c	n/a	n/a	in the past 20py	no
2875	m	69	Larynx	T1a	n/a	M0	n/a	35py	no
2876	m	55	Oropharynx	T2	N1	M0	p16+	quit at age of 34, 10py	2-4x/month
2877	m	63	Oropharynx	T3	N1	M0	p16+	never	2-4x/month
2993	m	59	Oropharynx	T4	N1	M0	p16+	in the past 25py	chronic abusus
3016	f	46	Larynx	T2	N0	M0	n/a	50py	no
3018	m	67	Oropharynx	T3	N2	M0	p16+	5py	no
3021	m	72	Hypopharynx	T2	N1	M0	n/a	in the past 60py	chronic abusus
3023	m	70	Larynx	T1	N0	M0	n/a	55py	n/a
3024	f	68	Oral cavity	T1	N0	M0	p16-	in the past	n/a
3026	m	66	Oral cavity	T2	Nx	M0	n/a	40py	no
3035	f	51	Oral cavity	T3	N0	M0	n/a	n/a	n/a
3043	f	78	Larynx	T1a	N0	M0	n/a	n/a	n/a
3044	m	58	Oral cavity	T4a	N2b	M0	neg	40 py	5-6/ week

3079	m	62	Larynx	T1a	N0	M0	xx	active	no
3082	m	61	Oropharynx	T4b	N1	M0	p16+	60py	no
3083	f	61	Oropharynx	T3	N1	M0	p16-	25py	2-4x/month
3108	m	58	Larynx	T3	N3b	M0	n/a	15py	2-3/ week wine
3111	m	66	Oropharynx	T1	N1	M0	p16+	40py	regulary
3118	m	67	Oral cavity	T1	N0	M0	n/a	40py	chronic abusus
3132	m	69	Larynx	T4a	N2b	M0	n/a	55py	> 4x/week
3134	m	84	Oropharynx	T2	N0	M0	n/a	in the past 50 py	no

Clinicopathologic characteristics of volunteers who participated in the study (immunofluorescence)

Patient #	Gender	Age at first diagnosis date	Tumor localisation	Tumor Stage	Nodal Status	Distant metastasis	HPV status	Smoking	Alcohol
239	m	63	Hypopharynx	T3	N2b	M0	n/a	n/a	n/a
127	m	55	Hypopharynx	T4	N0	M0	n/a	n/a	n/a
240	m	51	Larynx	T3	N2c	M0	n/a	n/a	n/a
141	m	64	Oropharynx	T2	N2b	M0	n/a	n/a	n/a
151	m	60	Hypopharynx	T2	N2a	M0	n/a	n/a	n/a
131	f	52	Hypopharynx	T4	N0	M0	n/a	n/a	n/a
241	m	63	Hypopharynx	T3	N2a	M0	n/a	n/a	n/a
132	m	57	Hypopharynx	T4	N0	M0	n/a	n/a	n/a
243	m	71	Oral cavity	T2	N2c	M0	n/a	n/a	n/a
203	f	42	Nasopharynx	T4	N2c	M0	n/a	n/a	n/a
146	m	60	Hypopharynx	T2	N2b	M0	n/a	n/a	n/a
146	m	60	Hypopharynx	T2	N2b	M0	n/a	n/a	n/a
296	m	67	Nasopharynx	T2	N1	M0	n/a	n/a	n/a
156	m	68	Hypopharynx	T2	N1	M0	n/a	n/a	n/a
235	m	65	Hypopharynx	T3	N2b	M0	n/a	n/a	n/a
244	m	69	Hypopharynx	T4	N0	M0	n/a	n/a	n/a

99	m	57	Hypopharynx	T4	N2b	M1	n/a	n/a	n/a
140	f	81	Hypopharynx	T4	N2c	M0	n/a	n/a	n/a
233	m	52	Oropharynx	T4	N0	M0	n/a	n/a	n/a
149	m	55	Hypopharynx	T4	N2a	M0	n/a	n/a	n/a
218	m	68	Hypopharynx	T4	N2c	M0	n/a	n/a	n/a
150	m	50	Hypopharynx	T4	N2c	M0	n/a	n/a	n/a
269	m	78	Larynx	T2	N0	M0	n/a	n/a	n/a
294	f	52	Nasopharynx	T2	N0	M0	n/a	n/a	n/a
232	m	61	Hypopharynx	T4	N2c	M0	n/a	n/a	n/a
89	m	54	Hypopharynx	T4	N2b	M0	n/a	n/a	n/a
201	m	62	Nasopharynx	T1	N2c	M0	n/a	n/a	n/a
142	m	57	Oropharynx	T4	N2c	Mx	n/a	n/a	n/a
237	m	69	Larynx	T4	N2b	M0	n/a	n/a	n/a
111	m	68	Hypopharynx	T1	N2b	M0	n/a	n/a	n/a
129	m	57	Hypopharynx	T4	N2a	M0	n/a	n/a	n/a
236	m	68	Hypopharynx	T4	N2b	M0	n/a	n/a	n/a
230	m	68	Hypopharynx	T4	N2c	M0	n/a	n/a	n/a
293	m	50	Nasopharynx	T4	N0	M0	n/a	n/a	n/a

Clinicopathologic characteristics of volunteers who participated in the study (Tumor killing assay)

**Patient #**	**Gender**	**Age at first diagnosis date**	**Tumor localisation**	**Tumor Stage**	**Nodal Status**	**Distant metastasis**	**HPV status**	**Smoking**	**Alcohol**
3108	m	58	Larynx	T3	N3b	M0	n/a	15py	2-3/ week wine
3100	m	58	Hypopharynx	T2	N0	M0	neg	30 pack per year	no
3110	m	64	Larynx	T3	N0	M0	xx	n/a	no
